# miR172b Controls the Transition to Autotrophic Development Inhibited by ABA in *Arabidopsis*

**DOI:** 10.1371/journal.pone.0064770

**Published:** 2013-05-23

**Authors:** Yanmin Zou, Youning Wang, Lixiang Wang, Lei Yang, Rui Wang, Xia Li

**Affiliations:** 1 Key State Laboratory of Plant Cell and Chromosome Engineering, Center of Agricultural Resources Research, Institute of Genetics and Developmental Biology, Chinese Academy of Sciences, Shijiazhuang, Hebei, China; 2 University of Chinese Academy of Sciences, Beijing, China; RIKEN Plant Science Center, Japan

## Abstract

Seedling establishment is a critical phase in the life of plants when they are the most vulnerable to environment. Growth arrest at post-germinative stage under stress is the major adaptive strategy to help germinating seedlings to survive a spectrum of stressful conditions. ABA signaling is the key pathway to control stress-induced developmental arrest. However, mechanisms controlling the phase transition under abiotic stress are not fully understood. Here, we described miR172b as a new key regulator controlling transition of germinating seedlings from heterotrophic to autotrophic growth under osmotic stress in *Arabidopsis*. We showed that miR172b and its target *SNZ* were co-expressed during early seedling development. Expression of miR172b and *SNZ* was low after radicle emergence and sharply increased at the checkpoint to autotrophic development under normal conditions. Interestingly, activation of miR172b and *SNZ* was completely abolished by ABA and osmotic stress. miR172b overexpression and *snz-1* exhibited increased sensitivity to ABA and osmotic stress during specific post-germinative stage, and resulted in higher expression of *ABI3*, *ABI5* and downstream genes, such as *Em6* and *RAB18*, than wild type under ABA treatment. Our results revealed that miR172b is a critical regulator specifically controlling cotyledon greening during post-germinative growth by directly targeting *SNZ* under ABA treatment and osmotic stress.

## Introduction

Seed germination, post-germinative growth and subsequent seedling establishment are central to successful stand establishment and population maintenance in plants, which are of great importance to agriculture and ecology [1]. The appearance of the radicle represents the end of germination and the beginning of the post-germinative stage, a period that ends when autotrophic growth is established. Over more than 100 years, extensive studies (> 25000 publications) have been made on the physiological and molecular mechanisms of seed germination using various plant species, including *Arabidopsis* [2]. Many key biological processes and regulators involved in metabolic regulation, hormonal regulation and molecular regulation have been identified [2]. In contrast, control of post-germinative growth and the phase transition from post-germinative growth to seedling establishment remain largely unclear, although they are as important as seed germination for successful plant establishment.

Compelling evidence has shown that the checkpoint from heterotrophic to autotrophic development at the post-germinative stage is genetically controlled, and that this phase transition is also highly vulnerable to various stresses including osmotic stress [2]. In order to prevent the adverse effects of environmental perturbations on the transition from post-germinative stage to subsequent seedling establishment, genetic and molecular mechanisms regulating adaptive response during the phase transition have evolved. It has been shown that ABA signaling plays key roles in both post-germinative growth arrest under stress and subsequent seedling establishment [1]. Mutations of the key components in the ABA signaling pathway, including receptors [3], protein phosphatase 2C (PP2C) family genes (*ABI1* and *ABI2*) [4]–[7], and transcription factors *ABI3* and *ABI5* [8], result in altered sensitivity to ABA during seed germination and the post-germinative stage [5], [9], [10]. Among these, *ABI3* and *ABI5* are expressed in a narrow developmental window and control the post-germination developmental arrest checkpoint. For example, the ABI5 level was undetectable on day 1 after stratification, but progressively increased thereafter and reached the highest level on day 4 [1]. Activation of ABI3 and ABI5 is required for maintaining the germinating seedlings at the post-germinative stage (embryonic state) to survive periods of severe drought stress [10].

microRNAs (miRNAs) are small, single-stranded, non-coding RNAs, which down-regulate their target genes at the post-transcriptional level through miRNA cleavage or translational repression [11], [12]. Several miRNAs have been linked with ABA responses and post-germination development. A prominent example in *Arabidopsis* is miR156, which regulates post-germinative development through modulating the mRNA of its target *SQUAMOSA PROMOTER-BINDING PROTEIN-LIKE13* (*SPL13*) [13]. Expression analysis suggests that this function of miR156 may be achieved through regulation of miR172 and *SCHNARCHZAPFEN* (*SNZ*), an *AP2*-like gene targeted by miR172 in *Arabidopsis* [14]. miR172 has been proved as a master regulator of the transition from vegetative growth to reproductive development [15], [16]. However, there are no reports to experimentally address the role of miR172 in stress tolerance. Here, we show that miR172b becomes induced at the checkpoint to autotrophic growth and controls the switch to autotrophic development in plants grown under normal conditions. Importantly, we report that miR172b failed to be turned on in the plants treated with ABA and osmotic stress to inhibit greening of cotyledon, and to prevent overly growth arrest from overexpression of *ABI3* and *ABI5* at post-germination stage. Further, we show that miR172b mediates its function by partially targeting *SNZ*. Together, these data identify miR172b as a master regulator in stress-induced developmental arrest and adaptation to stress.

## Materials and Methods

### Plant materials and growth conditions

Seeds of Columbia ecotype (Col-0), *35S: miR172b* [16], *snz-1* [17], *abi5-8* (SALK_013163) [18], *snz-1* overexpressing *SNZ* and *snz-1abi5-8* double mutants (Table S5) were surface-sterilized with 50% (v/v) bleach for 5 min and washed at least four times with sterile water. Sterile seeds were plated on MS medium plus 2% sucrose or the medium containing ABA (mixed isomers, Sigma), NaCl and mannitol. Plates were routinely kept for 2 days in the dark at 4°C to break dormancy (stratification) and transferred thereafter to a tissue culture room under constant light at 22°C with a 16/8 h light/dark regime.

### Vector construction and Arabidopsis transformation

To generate transgenic plants overexpressing *SNZ* under the control of cauliflower mosaic virus 35S promoter in *snz-1* mutant background, we cloned the coding region of *SNZ* by polymerase chain reaction (PCR) which was then inserted into the pTF101 vector containing a bar gene. The primers used for the construction are listed in Table S4. Transformation of Arabidopsis was performed using the floral dip method using Agrobacterium tumefaciens strain EHA105. Seeds were harvested and plated on the MS medium containing BASTA to identify transgenic plants. Lines containing single insertions were selected on the basis of the segregation ratio of 3∶1 and homozygous lines were used for the gene expression assay and phenotypic analysis.

### Genetic analysis

Homozygous *snz-1* was crossed with *abi5-8* to generate the *snz-1abi5-8* double mutant. Because both mutants contain T-DNA insertions, we performed PCR to identify the homozygous lines containing both mutations in *SNZ* and *ABI5* using the specific primers (LP1 and RP1, or RP1 and T-DNA left-border primer LBb1.3 for *abi5-8*; LP2 and RP2, or RP2 and LBb1.3 for *snz-1*). The primers used for genotyping are listed in Table S4.

### Germination and seedling development assays under ABA and osmotic stress treatments

For germination assays, at least 60 seeds from *35S: miR172b* [16], *snz-1* [17] and Col-0 plants were sown onto MS triplicate plates supplemented with 20 gL^−1^ sucrose, 8 gL^−1^ agar, and different concentrations of ABA (0, 0.2, 0.4, 0.8, 1 and 1.5 µM), NaCl (0, 50, 75, 100 and 150 mM), Mannitol (0, 100, 200, 300 and 400 mM). After 2-day stratification at 4°C in darkness, the plates were incubated in a growth chamber at 22±2°C under long-day conditions (16 h light/8 h dark). Germination was defined as the emergence of 1 mm or more of the radicle from the seed coat as described previously [19]. The degree of greening is defined as the extent of expanded cotyledons that are capable of turning green after transferred to light. The greening rate was expressed as the percentage of the germinating seedlings turning green.

All experiments were repeated three times with three replicates (n>60) in each. The data reported in the figures are means of the values with standard deviation (SD).

### Gene expression analysis

Seeds were kept in darkness at 4°C for 2 days with or without ABA (0.4 µM or 5 µM) and then transferred to constant light at 22°C. Total RNA was extracted from plant tissue using Trizol reagent (Invitrogen) according to the manufacturer's instructions. 2 mg of total RNA was DNase I-treated and single-stranded cDNA was synthesized using oligo (dT) and the RevertAid First Strand cDNA Synthesis Kit (Invitrogen). The sequences of primers that were used for gene expression analysis are listed in Table S3. Quantitative real-time PCR was run on a ABI Prism 7500 Sequence Detection System (Applied Biosystems, Foster City, CA) using the Platinum SYBR Green qPCR Supermix-UDG (Invitrogen). The expression level was normalized against the geometric mean of *GAPC* gene, as a reference gene expressed stably. All the experiments were performed three times, each with three replicates. Error bars denote SD.

### Electrophoretic mobility shift assays (EMSA)

Recombinant MBP-SNZ fusion protein was expressed and purified using Amylose Resin (NEB)according to the manufacturer's introduction. The binding activity of the protein was analyzed using an oligonucleotide containing 6 copies of RAV1-A motif, 5′-CACCTG(CAACA)_6_-3′ (wRAV1-A), labeled with biotin at the 3′ end (Invitrogen, USA). An oligonucleotide containing mutated RAV1-A motif, 5′-CACCTG(CGGTA)_ 6_-3′ (mRAV1-A), labeled with biotin at the 3′ end (Invitrogen, USA) was used as a control. The specific probes containing RAV1-A motifs corresponding to the fragment of *ABI5* promoter were synthesized and labeled with biotin at the 3′ end as mentioned above. The sequences of probes used for EMSA are listed in Table S4. EMSA was performed using LightShift® Chemiluminescent EMSA Kit according to the manufacturer's protocol. Complementary oligonucleotide pairs were annealed to make double-stranded and biotin-labeled probes (5 pmol) by mixing in a buffer (10 mM Tris and 1 mM EDTA), boiling for 5 min, and cooling slowly overnight. Unlabeled complementary oligonucleotide pairs were also annealed to make double-stranded competitor probes (50 and 250 times the amount of biotin-labeled *ABI5* probes). EMSA reaction solutions were prepared by adding the following components in order according to the manufacturer's protocol (LightShift Chemilu minescent EMSA kit): binding buffer, 50 ng poly (dI-dC), 2.5% glycerol, 0.06% NP-40, 5 mM MgCl_2_, 19 mg BSA, proteins (28 µg), competitor and biotin-labeled probes. Reaction solutions were incubated for 30 min at room temperature. The protein-probes mixture was separated in a 8% poly-acrylamide gel and transferred to a Biodyne B Nylon membrane. Migration of biotin-labeled probes was detected by using streptavidin-horseradish peroxidase conjugates that bind to biotin and chemiluminescent substrate according to the manufacturer's protocol. This experiment was performed three times.

### Statistic analysis

All the data were analyzed using software GraphPad Prism 5. The Student's t-test was performed and the statistically significant treatments were marked with ‘*’ (P<0.05), ‘**’ (P<0.01) and ‘***’ (P<0.001).

## Results

### miR172b controls the transition to autotrophic development and stress tolerance during osmotic stress

**Figure 1:**
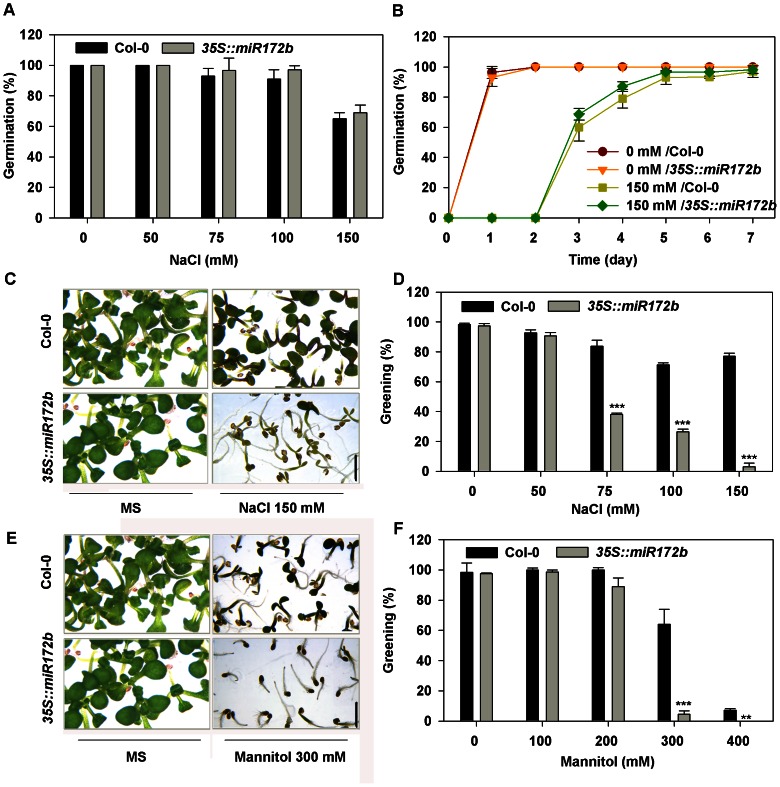
*35S:miR172b* transgenic plants are hypersensitive to salt and mannitol at post-germination stages. (A) Salt dose–response analysis of Col-0 and *35S:miR172b* during seed germination at 5 days after stratification. (B) A germination time course on medium containing 150 mM NaCl. (C) *35S: miR172b* plants showed delayed greening under salt treatments; Photographs were taken 7 days after stratification. Bar  = 5 mm. (D) Percentage of Col-0 and *35S: miR172b* turning green 7 days after stratification under salt. (E) Greening of *35S: miR172b* plants was more sensitive to mannitol; photographs were taken 5 days after stratification. Bar = 5 mm. (F) Percentage of Col-0 and *35S: miR172b* becoming green at 7 days after stratification under mannitol treatment. All the experiments were performed three times with three replicates. Error bars denote±SD. Student's t test was performed, and the statistically significant treatments are marked with asterisks. (*) P<0.05, (**) P<0.01 and (***) P<0.001.

After seed germination, germinating seedlings proceed through a critical developmental stage referred to as the transition from heterotrophic to autotrophic growth that can be tracked by cotyledon greening. Thus, cotyledon greening is thought to be the critical checkpoint in seedling establishment. When plant seeds are germinated under stress conditions, the developmental program to autotrophic growth is interrupted and stress reinstates the embryonic stage [2]. The resulting developmental arrest before autotrophic development is established could allow the germinating seedlings to maintain at quiescent state and survive a period of stress conditions [1]. As it has recently been shown that miR172 mediates post-germinative growth in *Arabidopsis* grown under normal conditions [13], we sought to determine whether miR172 may have a role in stress response and developmental checkpoint control under osmotic stress. To this end, stress response of plants overexpressing miR172b during early development was examined. No significant differences during seed germination were found between the miR172b overexpressors and the wild type under both normal conditions and salt stress (Figure 1A and B). However, with increasing concentrations of salt stress, miR172b overexpressors displayed a significant reduction in the rate of cotyledon greening compared with that of the wild type (Figure 1C and D). When miR172b overexpressors were exposed to moderate levels of salt (75 to 100 mM NaCl), a more than 50% reduction in greening rate of germinating miR172b overexpression seedlings was observed over a 7 day period. At higher level of NaCl (150 mM NaCl) that allowed about 75% of germinating wild type seedlings to turn green, very few (4% of total) of miR172b overexpressors turned green. No altered salt sensitivity was observed when greened miR172b overexpression seedlings grown under normal conditions were treated with salt stress (Figure S1A, B and E). Salt stress is caused by osmotic effects or ion effect [20]. To investigate whether the hypersensitivity of the miR172b overexpressors to salt is also caused by osmotic stress instead of ion-specific effect, we analyzed the response of 35S: miR172b transgenic plants under mannitol, LiCl and KCl treatments. The results showed that miR172b overexpressors also exhibited increased sensitivity in response to high concentrations of mannitol during early development (Figure 1E and F), but no significant differences were found during seed germination and greening under LiCl and KCl treatments (Figure S3). These data indicate that miR172b regulates general osmotic stress-induced developmental arrest and is specifically required for cotyledon greening, which is an indicator for the successful transition from heterotrophic to autotrophic growth under osmotic stress.

### miR172b mediates ABA induced-developmental arrest during the transition to autotrophic development

**Figure 2:**
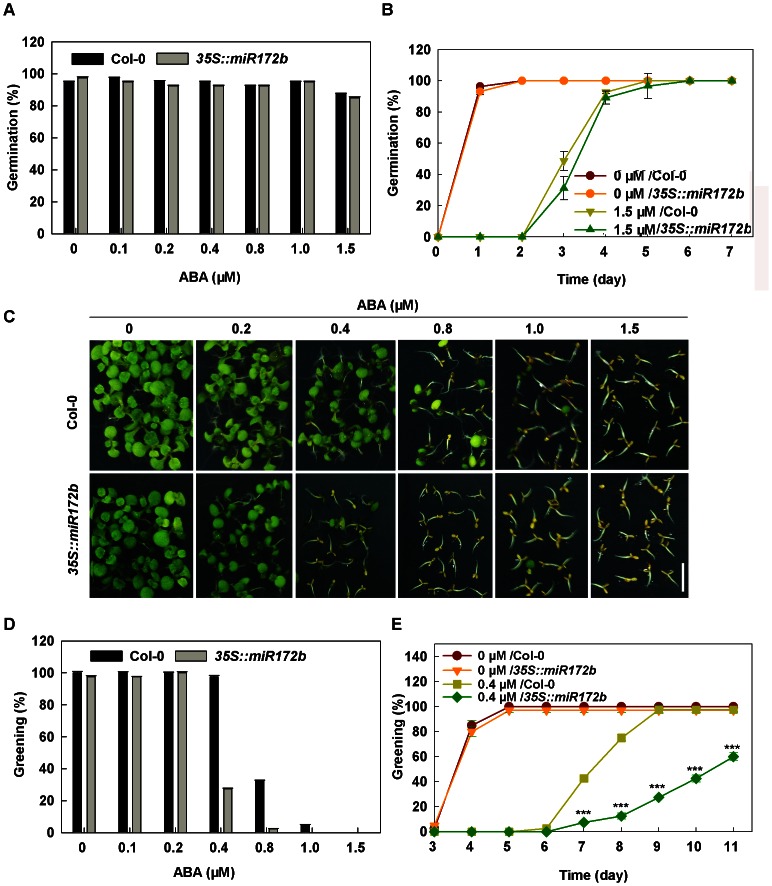
*35S: miR172b* plants are hypersensitive to ABA in post-germination stages. (A) ABA dose–response analysis of seed germination for Col-0 and *35S: miR172b* at 5 days after stratification. (B) A germination time course on medium containing 1.5 µM ABA. (C) *35S: miR172b* plants showed delayed greening under ABA treatments; photographs were taken 9 days after stratification. Bar = 5 mm. (D) ABA dose–response analysis of greening for Col-0 and *35S: miR172b* at 9 days after stratification. (E) A greening time course on medium containing 0.4 µM ABA. All the experiments were performed three times with three replicates. Error bars represent±SD. Student's t test was performed, and the statistically significant treatments are marked with asterisks. (*) P<0.05, (**) P<0.01 and (***) P<0.001.

Because ABA is previously shown to play a prominent role in regulation of post-germinative growth arrest under osmotic stress in *Arabidopsis* [21], [22], we examined the ABA response of miR172b overexpressors during germination and early development stages. We found that miR172b overexpression did not affect germination rate under normal conditions and ABA treatments (Figure 2A and B). Alteration in miR172b expression level also did not influence growth inhibition of the young seedlings caused by exogenous ABA after the transition from heterotrophic to autotrophic development was completed (Figure S1C and D). However, as expected, cotyledon greening of germinating seedlings overexpressing miR172b was dramatically affected when germinated on the medium containing various concentrations of ABA compared with that of the wild type (Figure 2C-E). When exposed to low concentrations of ABA (<0.4 µM), the germinating seedlings overexpressing miR172b displayed delayed greening, although their overall greening rate was the same as that of the wild type at the end of the seven day growing period (Figure 2C and D). In the presence of 0.4 µM ABA, the percentage of germinated seedlings overexpressing miR172b turning green was reduced by more than 70% during the growing period (Figure 2D). The timing for germinated miR172b overexpressors turning green was also dramatically postponed and the greening rate was lower than 30% when the wild type reached 100% on day 9 after stratification (Figure 2E). When germinated on a medium containing ABA concentrations greater than 0.8 µM, cotyledon greening of germinated miR172b overexpressors was completely blocked (Figure 2C and D). However, when the seedlings with yellowish cotyledons on 0.8 µM ABA were transferred onto MS medium, all of them turned green (Figure S2). Together, these results suggest that miR172b may regulate stress-induced developmental arrest and stress tolerance at the post-germinative/seedling establishment stage through the ABA signaling pathway.

### miR172b expression is down-regulated by osmotic stress and ABA

**Figure 3:**
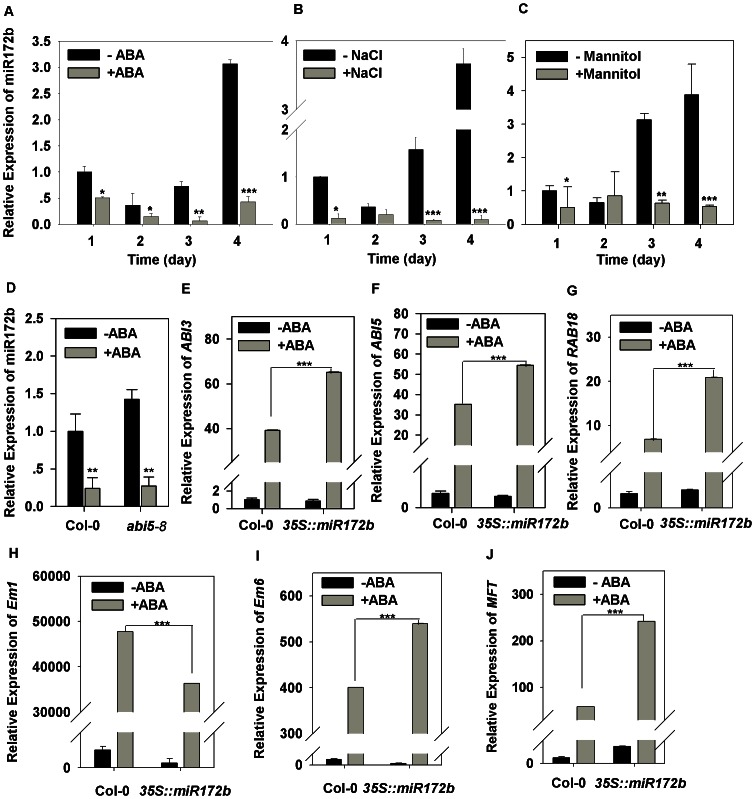
miR172b regulates post-germinative developmental arrest induced by abiotic stress through the ABA dependent pathway. Expression analysis of miR172b under ABA (5 µM) (A), NaCl (100 mM) (B) and mannitol (300 mM) (C) treatment in the first four days after stratification treatment in the first four days after stratification. (D) miR172b expression in wild-type (Col-0) and *abi5-8* treated without or with 0.4 µM ABA. Expression analysis of *ABI3* (E), *ABI5* (F), *RAB18* (G), *Em1* (H), *Em6* (I) and *MFT* (J) in germinating seeds of the wild type and *35S:miR172b* in the third day after stratification with or without 0.4 µM ABA. The expression level was normalized against the reference gene *GAPC*, and all the experiments were performed three times with three replicates. Error bars denote±SD. Student's t test was performed, and the statistically significant treatments are marked with asterisks. (*) P<0.05, (**) P<0.01 and (***) P<0.001.

As the levels of miR172 expression are critical for regulating phase transition during plant development, and overexpression of miR172b results in post-germinative developmental arrest under osmotic stress and ABA (Figure 1 and Figure 2), we therefore hypothesized that osmotic stress and ABA may regulate levels of miR172b expression to reprogram the developmental program allowing phase transition changes to occur. To this end, we first analyzed expression of miR172b in response to ABA and osmotic stress induced by NaCl treatment. In the absence of ABA, low levels of miR172b expression were detected in the first two days after stratification, and expression of miR172b decreased and reached its lowest level on day 2 (Figure 3A). miR172b expression then increased starting from day 3 after stratification and a sharp increase in its expression was detected on day 4 (Figure 3A). This observation of a sharp increase of miR172 is consistent with previous reports [16], [23], [24]. Activation of miR172b at the switch point suggests that miR172b plays a pivotal role in regulating the heterotrophic/autotrophic checkpoint during early development.

When wild type seeds were germinated on the medium containing 5 µM ABA, levels and patterns of miR172b expression were strikingly different (Figure 3A). In the first 2 days after stratification, the levels of miR172b expression were greatly down-regulated by ABA, although miR172b still maintained a downward trend. The decreasing trend in miR172b expression stopped with the lowest expression level on day 3. Most strikingly, we found that on day 4 after stratification, when miR172b was dramatically activated under normal conditions, miR172b expression after ABA treatment still remained at a very low level equal to that on day 2 after stratification under normal conditions. A very similar pattern of miR172b expression was observed of the wild type treated with osmotic stress (Figure 3B and C). These results suggest that down-regulation of miR172b expression by osmotic stress and ABA during early seedling development, in particular the total block of miR172b activation on day 4 after stratification, may contribute to developmental arrest at the post-germinative stage and heterotrophic/autotrophic transition failure under osmotic stress and ABA treatment.

As the expression of miR172b is negatively regulated by osmotic stress and ABA, we sought to determine whether the miR172b promoter contains any *cis* elements responsive to osmotic stress and/or ABA. Bioinformatic analysis of the miR172b promoter identified several ABA responsive *cis* elements, such as ABRE, Couple element 3 and *cis* elements related to stress response (e.g. MYB1, GAREAT and WRKY) in the promoter region of miR172b (Table S1) [25]–[28]. These data support a critical role for miR172b in control of transition to the autotrophic phase and in ABA-mediated stress tolerance during post-germination growth under osmotic stress.

### miR172b functions in ABA signaling during post-germinative development

Because *ABI3* and *ABI5* play a pivotal role in post-germinative arrest checkpoint control [9], [29], we then analyzed the relationship between miR172b and *ABI3* and *ABI5*. We found that the levels and patterns of miR172b expression were not significantly changed in *abi5-8* (Figure 3D) [18], a loss of function mutant showing decreased sensitivity to ABA inhibition. Next, we investigated whether the level of miR172b affects transcript level of *ABI3* and *ABI5* during the early developmental stage. Transcription levels of *ABI3* and *ABI5* were analyzed in *35S:miR172b* transgenic plants in the absence or presence of ABA. In the absence of ABA, *ABI3* and *ABI5* transcript levels of *35S:miR172b* transgenic plants were comparable to those of the wild type (Figure 3E and F). However, the induction levels of both *ABI3* and *ABI5* were significantly higher in the germinating plants overexpressing miR172b than those of the wild type in the presence of ABA. Expression of *ABI3* and *ABI5* increased 39-fold and 35-fold in the wild type at 3 day after ABA treatment, in sharp contrast, a 76-fold and 69-fold increase in *ABI3* and *ABI5* expression occurred by the time of ABA treatment in *35S:miR172b* transgenic plants (Figure 3E and F). It has been shown that overexpression of *ABI3* and *ABI5* greatly increases sensitivity to ABA and osmotic stress [10]. Therefore, it is possible that failure in miR172b activation at the heterotrophic/autotrophic transition point under ABA and osmotic stress may limit *ABI3* and *ABI5* overexpression and avoid triggering an overreaction of germination seedlings to stress during post-germinative development. To confirm the above hypothesis, we subsequently analyzed the expression of several ABA-responsive genes acting downstream of *ABI3* and *ABI5* during ABA-induced post-germinative growth arrest in the wild type and *35S:miR172b* transgenic line [30], [31]. Among them, *LEA* genes *Em1* and *Em6* are directly targeted by *ABI5* [30], and *RAB18* is regulated by *ABI5.* As shown in Figure 3G-I, all the genes were up-regulated after ABA treatment in the wild type, and the expression of *Em6* and *RAB18* was clearly up-regulated in *35S:miR172b* compared with wild type control. Previous study has shown that *MOTHER OF FT AND TFL1* (*MFT*) is also directly regulated by *ABI3* and *ABI5* during seed germination [32]. We found that *MFT* was induced by ABA at dramatically higher level in *35S:miR172b* germinating seedlings (Figure 3J). These results indicate that miR172b may regulate growth arrest of germinated embryos through *ABI3*/*ABI5*-dependent signaling pathway.

### *SNZ* was co-expressed with miR172b in the absence and presence of salt stress and ABA during early development

**Figure 4:**
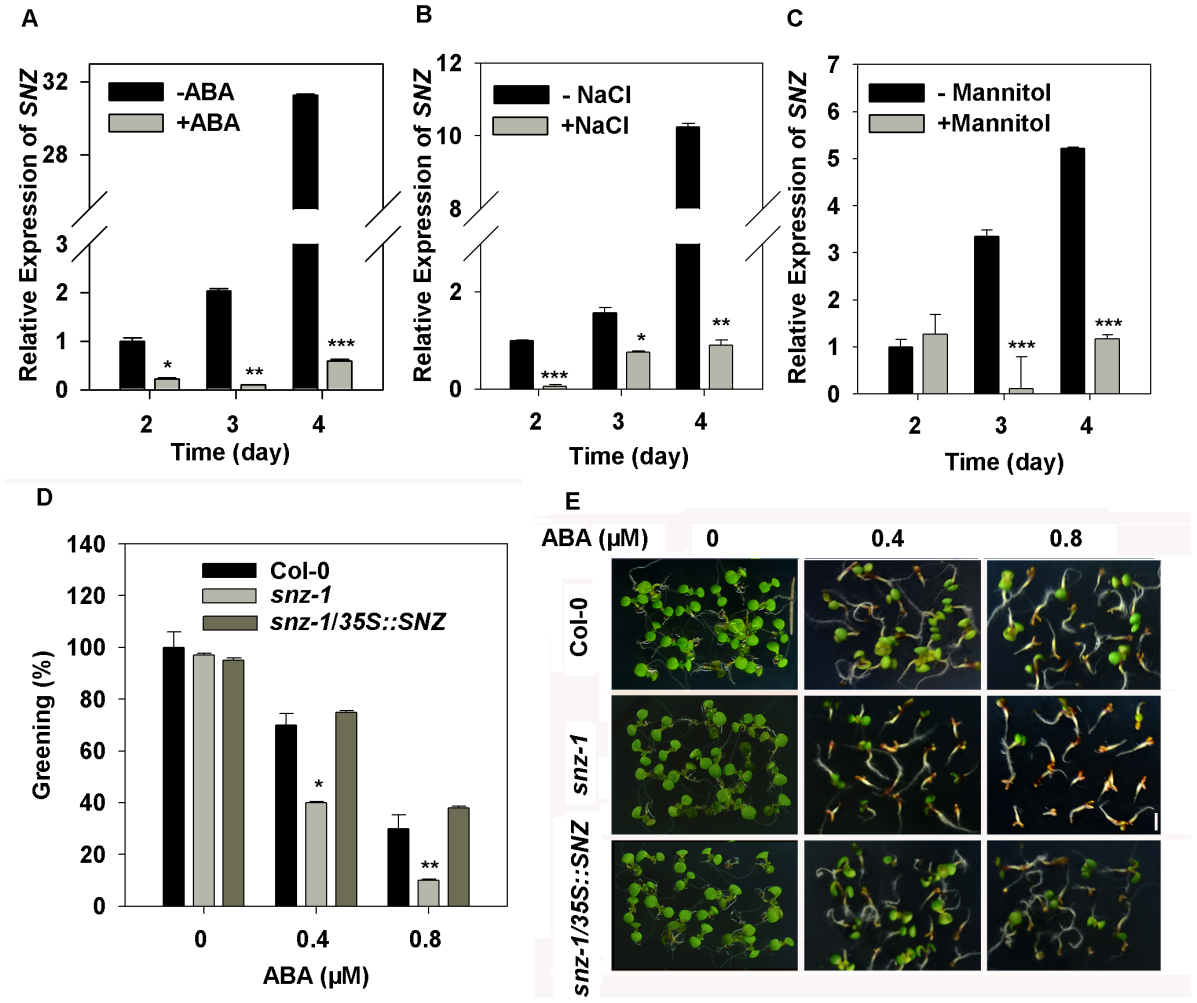
miR172b regulates post-germinative growth arrest by directly targeting *SNZ.* Wild-type seeds were germinated on MS with or without treatments, and materials were collected at 2 to 4 days after stratification. (A) *SNZ* expression in response to ABA (5 µM); (B) *SNZ* expression in response to NaCl (100 mM); (C) *SNZ* expression in response to mannitol (300 mM); (D) and (E) *snz-1* seedlings showed delayed greening under ABA treatments; Photographs were taken 7 days after stratification. Bar = 4 mm. All the experiments were performed three times with three replicates. Error bars denote±SD. Student's t test was performed, and the statistically significant treatments are marked with asterisks. (*) P<0.05, (**) P<0.01 and (***) P<0.001.The expression level was normalized against the reference gene *GAPC*. All the experiments were performed three times with three replicates. Error bars denote±SD.

In *Arabidopsis*, miR172 regulates developmental phase transition through targeting 6 *APETALA2-LIKE* (*AP2-like*) transcription factors: *AP2*, *TOE1*, *TOE2*, *TOE3*, *SMZ*, *SNZ* [14], [16], [23], [33]. We presumed that miR172b must regulate stress-induced growth arrest at the post-germinative stage through its target(s). To this end, we first searched the expression profiles of 6 target genes in response to osmotic stress and ABA from the public microarray database (http://bbc.botany.utoronto.ca/efp/cgi-bin/efpWeb.cgi). We found that among the targets, *SNZ* and *TOE3* were responsive to ABA and osmotic stress/drought in 7 and 18 day-old seedlings (Figure S4A and B). To verify whether *SNZ* and *TOE3* mediate osmotic stress/ABA-induced post-germinative growth arrest, we first analyzed the expression of *SNZ* and *TOE3* in response to ABA during post-germinative development in the wild type. As shown in Figure S5A, expression levels of *TOE3* on days 2 and 3 after stratification were low, and an approximately 8-10-fold increase in its expression was detected on day 4 regardless of whether or not they were treated with ABA. In sharp contrast, *SNZ* showed a quite similar expression pattern to that of miR172b. In the absence of ABA, the level of *SNZ* expression was the lowest on day 2 after stratification, then increased on day 3 and showed a sharp elevation on day 4 (Figure 4A). However, in the presence of ABA, expression of *SNZ* was greatly reduced, and remarkably, the sharp elevation of *SNZ* transcript on day 4 completely disappeared (Figure 4A). A similar pattern in *SNZ* expression was observed in the germinating seedlings under osmotic stress (Figure 4B and C). In addition, overexpression of miR172b greatly reduced the expression of *SNZ* under both normal conditions and ABA treatment, although the decreasing trend in its expression remained in response to ABA (Figure S5B). Moreover, promoter analysis revealed several ABA *cis* elements in the promoter region of *SNZ* (Table S2) [25], [26], [34]-[36]. Taken together, these results suggest that *SNZ* may function as a key target to regulate stress/ABA-induced post-germinative growth arrest. As compelling results have shown that miR172 generally co-expresses with its targets and regulates its target genes at a posttranslational level [23], [33], [37], it is conceivable that miR172b may also regulate stress/ABA-induced post-germinative growth arrest and stress tolerance by modulating SNZ at a posttranslational level.

### miR172b regulates stress/ABA-induced post-germinative growth arrest by partially targeting *SNZ*

To further prove whether *SNZ* is a functional target of miR172b during stress/ABA-induced post-germinative arrest, a loss-of-function mutant *snz-1* [17] was analyzed. No significant difference was observed between *snz-1* and the wild type during seed germination under normal conditions and ABA treatments (Figure S6B). In the absence of ABA, cotyledon greening and appearance of true leaves of *snz-1* germinating seedlings were comparable to the wild type (Figure 4D and E, Figure S6C). However, greening of the mutant seedlings was delayed in comparison with wild type in response to increasing concentrations of ABA (Figure 4D and E), suggesting that loss of function in *SNZ* affects the developmental arrest induced by ABA at a post-germinative stage. To prove the delayed greening phenotype of *snz-1* was due to mutation in the *SNZ*, we generated transgenic *snz-1* plants that express *SNZ* under control of the cauliflower mosaic virus 35S promoter. We isolated the independent transgenic lines showing similar level of *SNZ* expression to the wild type and found that *SNZ* expression in *snz-1* plants completely restored their ABA sensitive phenotype (Figure 4D, E, Figure S6A and C). This data confirm the role of *SNZ* during post-germinative development arrest induced by ABA.

However, when the *snz-1* mutant was compared with the *35S:miR172b* line, we found that miR172b overexpression plants displayed more severe phenotype in response to ABA (Figure 2E). These data indicate that *SNZ* may be just one of the genes targeted by miR172b to regulate ABA-dependent developmental arrest during the post-germinative stage. Based on ABA induction of *TOE3,* we speculate that *TOE3* may be another potential target of miR172b in this process.

### miR172b-*SNZ* module functions upstream of *ABI5* in regulating stress/ABA-induced post-germinative growth arrest

**Figure 5:**
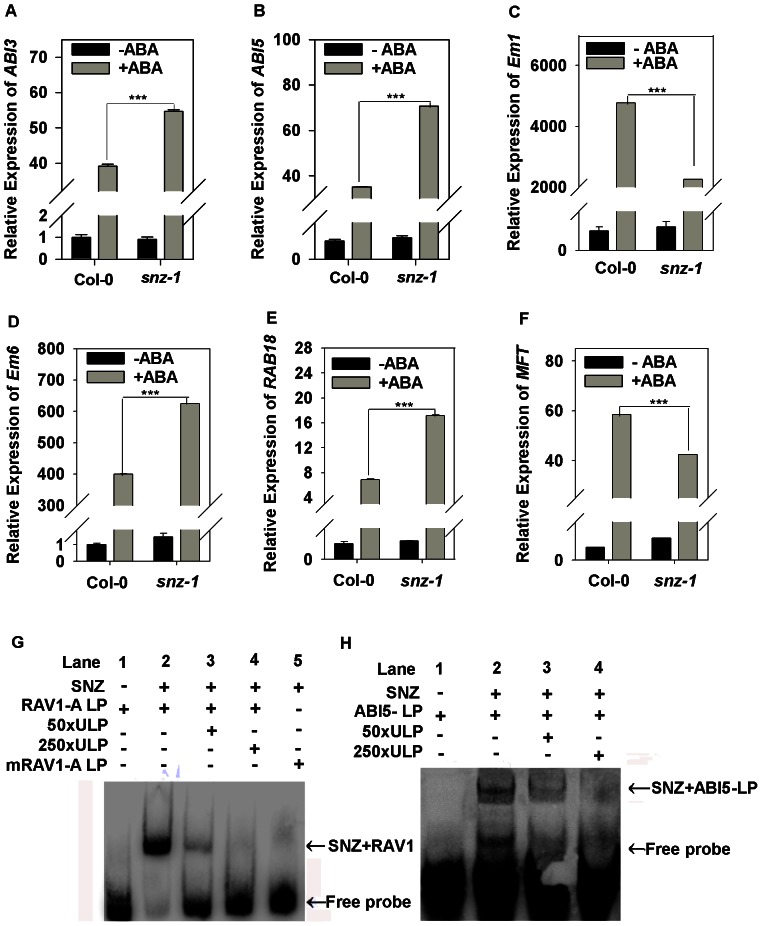
miR172b regulates post-germinative growth arrest through modulating the expression of *ABI3* and *ABI5*. Expression of *ABI3* (A), *ABI5* (B), *Em1* (C), *Em6* (D), *RAB18* (E) and *MFT* (F) in the wild type and *snz-1* seeds on day 3 after stratification with or without 0.4 µM ABA. The expression level was normalized against the reference gene *GAPC*. Student's t test was performed, and the statistically significant treatments are marked with asterisks. (*) P<0.05, (**) P<0.01 and (***) P<0.001. (G) SNZ interacts with RAV1 motif. Recombinant MBP-SNZ was used in an EMSA with biotin-labeled oligonucleotide containing 6 copies of the RAV1 motif as the probe; and the probe with the mutated RAV1 (mRAV1) was used as a negative control; (H) SNZ interaction with ABI5 promoter fragment containing RAV1 motif. Unlabeled probes were used as competitors to test the binding specificity. Arrows heads indicate the positions of protein-DNA probe complex and free probes, respectively. All the experiments were performed three times with three replicates, and the representative result is shown. Error bars denote±SD.

As overexpression of miR172b affected transcript levels of *ABI3* and *ABI5* during post-germinative development, we predicted that loss of function in *SNZ* during early development would result in a similar expression pattern of these genes if *SNZ* is a direct target of miR172b in the process. To test this, expression of these genes in germinating *snz-1* seedlings with and without ABA treatment was analyzed. As expected, *ABI3* and *ABI5* transcript levels were markedly increased (Figure 5A and B). Notably, the relative increase in *ABI3* and *ABI5* expression in the *snz-1* mutant was lower than that in the miR172b overexpressor (Figure 3E and F, Figure 5A and B). We then examined the expression patterns of *ABI5*-regulated genes, all the genes we examined were up-regulated by ABA in both of the *snz-1* mutant and wild type (Figure 5C-F). Among these genes, *Em6* and *RAB18* expression was also greatly up-regulated by ABA in *snz-1* mutant (Figure 5D and E) compared with wild type, which is consistent with the expression pattern in plants overexpressing miR172b (Figure 3G and I). However, *MFT* expression was down-regulated in *snz-1* mutant compared with the wild type, although it was still able to be induced by ABA (Figure 5F). This result indicates that miR172b may regulate post-germinative growth arrest by targeting *SNZ* via an *ABI3*/*ABI5* dependent pathway.

*SNZ* and other target genes of miR172b encode AP2-type transcription factors. We assumed that these transcription factors might bind to the promoters of ABA-responsive genes to regulate ABA signaling and response. To this end, we analyzed a 2-kb upstream region of a putative transcription start code for the ABA-response genes. Interestingly, 7 to 13 RAV1-A (CAACA) *cis* regulatory elements [38], which are the binding sites of AP2-type transcription factors, in the promoter regions of *ABI3*, *ABI5*, and their downstream genes *Em1*, *Em6*, *RAB18* and *MFT* were identified (Table S6).

To analyze binding activity of SNZ to RAV1-A motif (CAACA) detected in Table S6, we performed electrophoretic mobility shift assays (EMSA) using biotin labeled probe containing RAV1-A motif and MBP-SNZ fusion protein. As shown in Figure 5G, the control lane with only DNA probe showed a single band corresponding to the unbound DNA fragment. However, after adding SNZ protein to the reaction, DNA-SNZ interaction was clearly detected (Figure 5G). By contrast, addition of unlabeled RAV1-A probe resulted in reduced binding activity of MBP-SNZ to the labeled RAV1-A containing oligonucleotide (Figure 5G). To verify the specificity between SNZ protein and RAV1-A motif containing DNA fragment, we replaced the RAV1-A containing probe with a mutated DNA probe (CAACA→CGGTA), and no SNZ-DNA complex was observed. These results indicate that SNZ protein can specifically bind RAV1-A motifs. To determine the physical interactions between SNZ and *ABI5* promoters, an EMSA was performed using 31 nt (-1 to -31) promoter fragments of *ABI5*, which contain the RAV1-A motif, as probes. The results showed that SNZ directly interacted with *ABI5* promoter fragment (Figure 5H). By contrast, addition of unlabeled probes inhibited the interactions. The result confirms that SNZ binds to *ABI5* promoter, suggesting that SNZ may directly regulate expression of *ABI5*.

**Figure 6:**
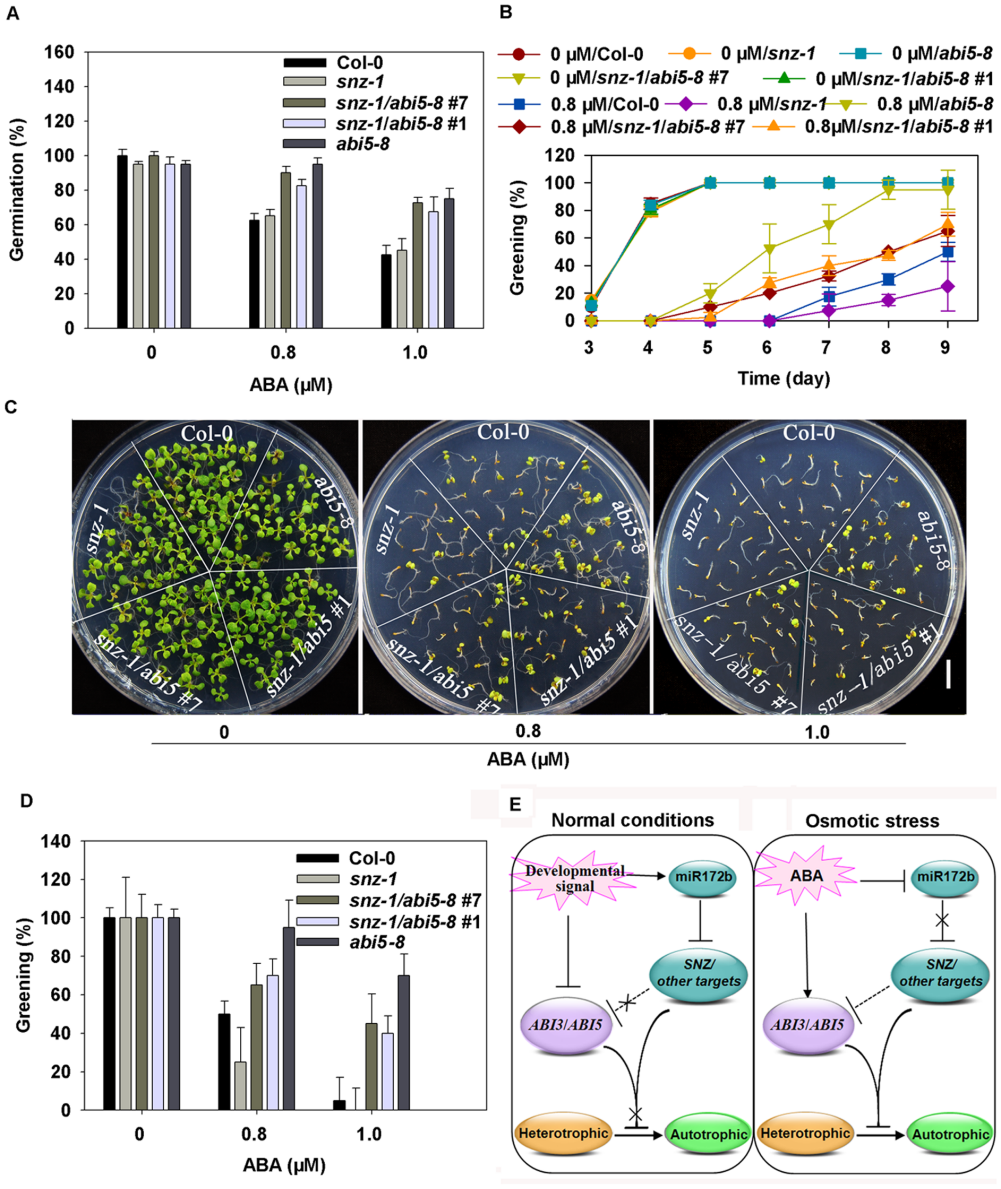
Epistatic analysis of relationship between *SNZ* and *ABI5* and a working model for post-germinative growth arrest induced by ABA. (A) ABA dose–response analysis of Col-0, *snz-1*, *snz-1*/*abi5-8* #7, *snz-1*/*abi5-8* #1 and *abi5-8* during seed germination. (B) A greening time course on medium containing 0.8 µM ABA. (C) *abi5-8* mutation is epistatic to *snz-1* mutation in ABA-induced inhibition of post-germinative growth; photographs were taken 9 days after stratification. Bar = 10 mm. (D) Greening on medium containing 0, 0.8 and 1.0 µM ABA. All the experiments were performed three times with three replicates. Error bars denote±SD. (E) A hypothetical model for the role of miR172b in controlling phase transition from heterotrophic to autotrophic growth.

To further prove the genetic relationship between SNZ and ABI5, we performed an epistatic assay for *snz-1* and *abi5-8*. We generated a *snz-1abi5-8* double mutant and performed phenotypic analysis. As shown in Figure 6, the *snz-1abi5-8* double mutant exhibited ABA insensitive phenotypes similar to the *abi5-8* mutant during both germination (Figure 6A) and cotyledon greening stages (Figure 6B, C, D). The result demonstrates that *ABI5* functions downstream of *SNZ* in the ABA signaling pathway and plant response.

## Discussion

Plants have two distinct developmental stages: heterotrophic and autotrophic development. Transition from the heterotrophic to autotrophic stage is a key step for plants to become autotrophs and complete their life cycle [39]. As sessile plants have to cope with an adverse environment after germination, it is very important for germinating seedlings to be sensitive to unfavorable conditions and be able to become arrested at the post-germinative growth stage in order to successfully survive a period of stress [40]–[42]. Therefore, transition to the autotrophic phase under various growth conditions must be fine-tuned and strictly regulated. Previous studies have revealed the critical role of the ABA signaling pathway in controlling post-germinative growth arrest and stress tolerance [1], [10], but the molecular mechanism is not fully understood. Here, we identified miR172b as a key molecule that regulates the checkpoint between heterotrophic and autotrophic growth under both normal and stress conditions.

It has been shown that increased expression level of miR172 determines the transition from vegetative growth to reproductive development [23], [33]. In this study, we found that miR172b is dynamically expressed during the post-germination stage, and sharp activation of miR172b at the switch point to the autotrophic stage (Figure 3 A-C) may determine cotyledon greening and subsequent success of the phase transition to phototrophy under normal conditions. Our results that *35S:miR172b* showed normal cotyledon greening under normal conditions (Figure 2C-E) support the above hypothesis. The question remaining to be answered is whether miR172b is essential for cotyledon greening under normal condition, and analysis of mutations in miR172b will provide direct evidence. The previous report has shown that the mutated *SPL13*, a target gene of miR156, led to increased expression of miR172a and miR172b and subsequent delayed appearance of the first pair of true leaves under normal conditions [13], but cotyledon greening and expansion. This observation is also favor of the hypothesis that high levels of miR172b are required for normal cotyledon greening. Seed germination, cotyledon greening and emergence of true leaves are fine-tuned processes regulated by different signaling pathways. It is likely that levels of miR172a and miR172b expression under control of miR156 and its target *SPL13* regulate appearance of first pair of true leaves under normal conditions.

When plants are subjected to osmotic stress, osmotic stress-induced modification of miR172b expression, in particular the failure of sharp activation at the switch to the autotrophic mode (Figure 6 E), may reinstate the embryonic program and metabolism, thus preventing damage and death in response to osmotic stress. Under osmotic stress, activation of the ABA signaling pathway is crucial for post-germination growth arrest by induction of *ABI3* and *ABI5*, with maximum expression level on day 4 after stratification. Activation of *ABI3* and *ABI5* and their level control are necessary for survival of germinating seedlings under stress and regrowth after stress removal. Indeed, overexpression of *ABI3* and *ABI5* results in phenotypes displaying more severe arrest in response to osmotic stress or ABA [10]. Inhibition of miR172b activation on day 4 after stratification by ABA may function as a positive regulator to control the level of *ABI3* and *ABI5* under osmotic stress, thus protecting germinating young seedlings from excessive stress. Indeed, overexpression of miR172b results in higher levels of *ABI3* and *ABI5* and hypersensitivity in response to ABA and stress treatments (Figure 1C and E, Figure 2C, Figure 3E and F). Most importantly, our results demonstrate that SNZ has a sequence-specific DNA-binding activity and can bind to the *ABI5* promoter region harboring RAV1 motif (Figure 5H). Further genetic evidence confirmed that *SNZ* functions upstream of *ABI5* and negatively regulates *ABI5* and the ABA signaling (Figure 5B-F, Figure 6A-D). The data that overexpression of miR172b results in substantial increases in expression levels of *Em6*, *MFT* and *RAB18*, downstream components of *ABI3* and *ABI5* also support the above hypothesis (Figure 3G-J) [30], [32], [43]. Our findings suggested that SNZ is involved in transcriptional regulation of ABI5 in ABA mediated plant response to osmotic stress during post-germinative development and seedling establishment. Previously, it has been shown that MFT represses *ABI5* transcription during seed germination[32]. Since MFT protein does not have any DNA binding domain, it has been proposed that it may regulate *ABI5* expression through other transcription factors[32]. Here we demonstrate a transcription factor SNZ physically binds the *ABI5* promoter to negatively regulate its expression. It remains to be determined whether SNZ is involved in guiding MFT to the *ABI5* promoter and corporately modulating *ABI5* transcription level.

Post-germinative development is a complex biological process, and successful phase transition from heterotrophic to autotrophic growth involves the complex interplay of the developmental and stress response signaling pathways and precise regulation at multiple levels. miRNA has been considered as fine-tuned regulators of the expression levels of their targets and the related biological processes. It is apparent that miR172b-*SNZ* is an important regulatory module in regulation of *ABI5* expression and subsequent post-germinative development of plants in response to abiotic stress. However, we do not exclude the possibility that miR172b may achieve its regulatory function by targeting multiple genes, because loss of function in *SNZ* alone exhibited less severe phenotypes compared with miR172b overexpressing plants in response to ABA (Figure 2C-E, Figure 4D, E).

Our studies reveal that miR172b may have evolved to fine-tune adaptive regulation of the heterotrophic to autotrophic checkpoint and developmental remodeling in response to adverse environmental conditions in *Arabidopsis*. However, many questions remain unknown. How is the ABA signal perceived and transmitted to miR172b? How do the multiple target genes of miR172b fine-tuning the post-germinative development and adaptation? Does SNZ function as a MFT interacting partner to repress *ABI5* expression? Answering these questions in future research will provide novel insight into the molecular mechanisms underlying the miR172b-*SNZ* mediated ABA signal transduction during post-germinative development stage in response to abiotic stress.

## Supporting Information

Figure S1 **Phenotypic analysis of young seedlings of** ***35S::miR172b*** **and Col-0 in response to ABA and NaCl.** The seeds of *35S::miR172b* and Col-0 were germinated on MS medium for 5 days and then transferred to MS medium containing NaCl (0, 50, 100 and 150 mM) or ABA (0, 50 and 100 µM). The photographs were taken 7 days after transfer to NaCl (A), and the primary root length was estimated (B). Photographs were taken 5 days after transfer to ABA (C), and the primary root length was estimated (D). (E) Identification of overexpression of miR172b in *35S:miR172b* transgenic lines. Bar = 250 µm. All the experiments were performed three times with three replicates. Bars represent mean±SD.
(TIF)

Figure S2 **Post-geminative developmental arrest of** ***35S:: miR172b*** **on ABA was restored after removing ABA.** Seeds of *35::miR172b* and Col-0 were germinated on MS medium containing 0.8 µM ABA, the seedlings which turned green were counted at 12 days after stratification (A); the arrested seedlings were transferred onto MS medium (B) or MS plus 0.8 µM ABA (C) for another 3 days, and the number of seedlings turning green were recorded. Bar =  5mm. All the experiments were performed three times with three replicates. Bars represent mean±SD.
(TIF)

Figure S3 **Phenotype of** ***35::miR172b*** **transgenic plants and** ***snz-1*** **under LiCl and KCl treatment.** (A) KCl and LiCl dose–response analysis of seed germination for Col-0, *35S:: miR172b* and *snz-1* at 3 days after stratification. (B) and (C) Greening of *35:: miR172b* transgenic plants and *snz-1* was not affected under KCl and LiCl treatment. Photographs were taken 7 days after stratification. Bar = 5 mm. All the experiments were performed three times with three replicates. Error bars denote±SD.
(TIF)

Figure S4 ***SNZ*** **and** ***TOE3*** **were up-regulated by ABA and osmotic stress/drought.** Plant materials from 7 and 18 day old wild-type Col-0 was treated by ABA and osmotic stress, respectively. (A) and (B) Expression of *SNZ* (A) and *TOE3* (B) in response to ABA and osmotic stress/drought; The colors from yellow to red indicate the increased absolute signal values of *SNZ* and *TOE3* expression retrieved from microarray data. (http://bbc.botany.utoronto.ca/efp/cgi-bin/efpWeb.cgi).
(TIF)

Figure S5 **Expression of** ***SNZ*** **and** ***TOE3*** **in response to ABA.** (A) Expression analysis of *TOE3* in response to ABA. Wild-type seeds were germinated on MS with or without 5 µM, and materials were collected at 2 to 4 days after stratification. (B) *SNZ* expression in wild-type (Col-0) and *35S:: miR172b* plants on day 3 and 4 after stratification with or without 0.4 µM ABA. Student's t test was performed, and the statistically significant treatments are marked with asterisks. (*) P<0.05, (**) P<0.01 and (***) P<0.001. All the experiments were performed three times with three replicates. Error bars denote±SD.
(TIF)

Figure S6 ***SNZ*** **regulates post-germinative developmental arrest induced by abiotic stress through the ABA dependent pathway.** *SNZ* cDNA under control of 35S promoter was expressed in *snz-1* mutant, and the transgenic lines were characterized and used for phenotypic analysis. (A) Transcript abundance of *SNZ* in wild-type, *snz-1* and *35S:: SNZ*/*snz-1* transgenic lines was monitored using RT-PCR. Shown are RT-PCR products amplified after 30 cycles, and the transcript expression levels were normalized against the reference gene *GAPC*. The red boxes indicate the line which was used in the phenotypic analysis. (B) ABA dose–response analysis of seed germination for Col-0 and *snz-1* at 5 days after stratification. (C) A greening time course on medium containing 0.4 µM ABA. All the experiments were performed three times with three replicates. Error bars denote±SD.
(TIF)

Figure S7 **Genotyping of** ***snz-1*** **/** ***abi5-8*** **.** (A) Schematics showing the location of the T-DNA insertion in *abi5-8*(SALK_013163) and *snz-1*(SALK_030031). Black boxes indicate exons, green box indicate 5′UTR, yellow box indicates 3′UTR, and the horizontal lines indicate introns. (B) Confirmation of the homologous T-DNA insertion in *snz-1/abi5-8* by PCR. Col-0 was used as positive control. The red boxes indicate the lines with the homologous T-DNA insertions both *snz-1* and *abi5-8* mutations, which were used in the phenotypic analysis.
(TIF)

Table S1 **cis-acting regulatory elements analysis of promoter sequence of miR172b by PLACE and PROMO.**
(DOC)

Table S2 **cis-acting regulatory elements analysis of the SNZ promoter sequence by PLACE and AtcisDB.**
(DOC)

Table S3 **Primers pairs used for real-time RT-PCR (Sequence 5′→3′).**
(DOC)

Table S4 **Primers pairs used for construction and genotyping, probes used for EMSA (Sequence 5′→3′).**
(DOC)

Table S5 **Information of the plant materials used in this work.**
(DOC)

Table S6 **cis-acting regulatory elements RAV1 analysis of promoter sequence of ABA response genes by PLACE and AtcisDB.**
(DOC)

## References

[1] Lopez-Molina L, Mongrand S, Chua NH (2001) A postgermination developmental arrest checkpoint is mediated by abscisic acid and requires the ABI5 transcription factor in Arabidopsis. Proc Natl Acad Sci U S A 98: 4782–4787. 11287670 10.1073/pnas.081594298 PMC31911

[2] Rajjou L, Duval M, Gallardo K, Catusse J, Bally J, et al (2012) Seed germination and vigor. Annu Rev Plant Biol 63: 507–533. 22136565 10.1146/annurev-arplant-042811-105550

[3] Park SY, Fung P, Nishimura N, Jensen DR, Fujii H, et al (2009) Abscisic acid inhibits type 2C protein phosphatases via the PYR/PYL family of START proteins. Science 324: 1068–1071. 19407142 10.1126/science.1173041 PMC2827199

[4] Fujii H, Chinnusamy V, Rodrigues A, Rubio S, Antoni R, et al (2009) In vitro reconstitution of an abscisic acid signalling pathway. Nature 462: 660–664. 19924127 10.1038/nature08599 PMC2803041

[5] Gosti F, Beaudoin N, Serizet C, Webb AAR, Vartanian N, et al (1999) ABI1 protein phosphatase 2C is a negative regulator of abscisic acid signaling. Plant Cell 11: 1897–1909. 10521520 10.1105/tpc.11.10.1897 PMC144098

[6] Umezawa T, Sugiyama N, Mizoguchi M, Hayashi S, Myouga F, et al (2009) Type 2C protein phosphatases directly regulate abscisic acid-activated protein kinases in Arabidopsis. Proc Natl Acad Sci U S A 106: 17588–17593. 19805022 10.1073/pnas.0907095106 PMC2754379

[7] Soon FF, Ng LM, Zhou XE, West GM, Kovach A, et al (2012) Molecular mimicry regulates ABA signaling by SnRK2 kinases and PP2C phosphatases. Science 335: 85–88. 22116026 10.1126/science.1215106 PMC3584687

[8] Nambara E, Okamoto M, Tatematsu K, Yano R, Seo M, et al (2010) Abscisic acid and the control of seed dormancy and germination. **Seed Sci RES** 20: 55–67.

[9] Koornneef M, Reuling G, Karssen CM (1984) The Isolation and Characterization of Abscisic-Acid Insensitive Mutants of Arabidopsis-Thaliana. **Physiol PlantarumLANTARUM** 61: 377–383.

[10] Lopez-Molina L, Mongrand S, McLachlin DT, Chait BT, Chua NH (2002) ABI5 acts downstream of ABI3 to execute an ABA-dependent growth arrest during germination. Plant J 32: 317–328. 12410810 10.1046/j.1365-313x.2002.01430.x

[11] He L, Hannon GJ (2004) MicroRNAs: small RNAs with a big role in gene regulation. Nat Rev Genet 5: 522–531. 15211354 10.1038/nrg1379

[12] Valencia-Sanchez MA, Liu J, Hannon GJ, Parker R (2006) Control of translation and mRNA degradation by miRNAs and siRNAs. Genes Dev 20: 515–524. 16510870 10.1101/gad.1399806

[13] Martin RC, Asahina M, Liu PP, Kristof JR, Coppersmith JL, et al (2010) The microRNA156 and microRNA172 gene regulation cascades at post-germinative stages in Arabidopsis. **Seed Sci RES** 20: 79–87.

[14] Schmid M, Uhlenhaut NH, Godard F, Demar M, Bressan R, et al (2003) Dissection of floral induction pathways using global expression analysis. Development 130: 6001–6012. 14573523 10.1242/dev.00842

[15] Lauter N, Kampani A, Carlson S, Goebel M, Moose SP (2005) microRNA172 down-regulates glossy15 to promote vegetative phase change in maize. Proc Natl Acad Sci U S A 102: 9412–9417. 15958531 10.1073/pnas.0503927102 PMC1166634

[16] Wu G, Park MY, Conway SR, Wang JW, Weigel D, et al (2009) The sequential action of miR156 and miR172 regulates developmental timing in Arabidopsis. Cell 138: 750–759. 19703400 10.1016/j.cell.2009.06.031 PMC2732587

[17] Mathieu J, Yant LJ, Murdter F, Kuttner F, Schmid M (2009) Repression of flowering by the miR172 target SMZ. PLoS Biol 7: e1000148. 19582143 10.1371/journal.pbio.1000148 PMC2701598

[18] Zheng Y, Schumaker KS, Guo Y (2012) Sumoylation of transcription factor MYB30 by the small ubiquitin-like modifier E3 ligase SIZ1 mediates abscisic acid response in Arabidopsis thaliana. Proc Natl Acad Sci U S A 109: 12822–12827. 22814374 10.1073/pnas.1202630109 PMC3411956

[19] Lu C, Hills MJ (2002) Arabidopsis mutants deficient in diacylglycerol acyltransferase display increased sensitivity to abscisic acid, sugars, and osmotic stress during germination and seedling development. Plant Physiol 129: 1352–1358. 12114588 10.1104/pp.006122 PMC166528

[20] Sumer A, Zorb C, Yan F, Schubert S (2004) Evidence of sodium toxicity for the vegetative growth of maize (Zea mays L.) during the first phase of salt stress. J Appl Bot Food Qual 78: 135–139.

[21] Wang ZY, Xiong L, Li W, Zhu JK, Zhu J (2011) The plant cuticle is required for osmotic stress regulation of abscisic acid biosynthesis and osmotic stress tolerance in Arabidopsis. Plant Cell 23: 1971–1984. 21610183 10.1105/tpc.110.081943 PMC3123942

[22] Xiong L, Zhu JK (2003) Regulation of abscisic acid biosynthesis. Plant Physiol 133: 29–36. 12970472 10.1104/pp.103.025395 PMC523868

[23] Aukerman MJ, Sakai H (2003) Regulation of flowering time and floral organ identity by a MicroRNA and its APETALA2-like target genes. Plant Cell 15: 2730–2741. 14555699 10.1105/tpc.016238 PMC280575

[24] Huijser P, Schmid M (2011) The control of developmental phase transitions in plants. Development 138: 4117–4129. 21896627 10.1242/dev.063511

[25] Higo K, Ugawa Y, Iwamoto M, Korenaga T (1999) Plant cis-acting regulatory DNA elements (PLACE) database: 1999. Nucleic Acids Res 27: 297–300. 9847208 10.1093/nar/27.1.297 PMC148163

[26] Prestridge DS (1991) Signal Scan - a Computer-Program That Scans DNA-Sequences for Eukaryotic Transcriptional Elements. Computer Applications in the Biosciences 7: 203–206. 2059845 10.1093/bioinformatics/7.2.203

[27] Messeguer X, Escudero R, Farre D, Nunez O, Martinez J, et al (2002) PROMO: detection of known transcription regulatory elements using species-tailored searches. Bioinformatics 18: 333–334. 11847087 10.1093/bioinformatics/18.2.333

[28] Farre D, Roset R, Huerta M, Adsuara JE, Rosello L, et al (2003) Identification of patterns in biological sequences at the ALGGEN server: PROMO and MALGEN. Nucleic Acids Res 31: 3651–3653. 12824386 10.1093/nar/gkg605 PMC169011

[29] Finkelstein R, Gampala SS, Lynch TJ, Thomas TL, Rock CD (2005) Redundant and distinct functions of the ABA response loci ABA-INSENSITIVE(ABI)5 and ABRE-BINDING FACTOR (ABF)3. Plant Mol Biol 59: 253–267. 16247556 10.1007/s11103-005-8767-2

[30] Carles C, Bies-Etheve N, Aspart L, Leon-Kloosterziel KM, Koornneef M, et al (2002) Regulation of Arabidopsis thaliana Em genes: role of ABI5. Plant J 30: 373–383. 12000684 10.1046/j.1365-313x.2002.01295.x

[31] Nakamura S, Lynch TJ, Finkelstein RR (2001) Physical interactions between ABA response loci of Arabidopsis. Plant J 26: 627–635. 11489176 10.1046/j.1365-313x.2001.01069.x

[32] Xi W, Liu C, Hou X, Yu H (2010) MOTHER OF FT AND TFL1 regulates seed germination through a negative feedback loop modulating ABA signaling in Arabidopsis. Plant Cell 22: 1733–1748. 20551347 10.1105/tpc.109.073072 PMC2910974

[33] Chen X (2004) A microRNA as a translational repressor of APETALA2 in Arabidopsis flower development. Science 303: 2022–2025. 12893888 10.1126/science.1088060 PMC5127708

[34] Yilmaz A, Mejia-Guerra MK, Kurz K, Liang X, Welch L, et al (2011) AGRIS: the Arabidopsis Gene Regulatory Information Server, an update. Nucleic Acids Res 39: D1118–1122. 21059685 10.1093/nar/gkq1120 PMC3013708

[35] Palaniswamy SK, James S, Sun H, Lamb RS, Davuluri RV, et al (2006) AGRIS and AtRegNet. A platform to link cis-regulatory elements and transcription factors into regulatory networks. Plant Physiol 140: 818–829. 16524982 10.1104/pp.105.072280 PMC1400579

[36] Davuluri RV, Sun H, Palaniswamy SK, Matthews N, Molina C, et al (2003) AGRIS: Arabidopsis gene regulatory information server, an information resource of Arabidopsis cis-regulatory elements and transcription factors. BMC Bioinformatics 4: 25. 12820902 10.1186/1471-2105-4-25 PMC166152

[37] Zhu QH, Upadhyaya NM, Gubler F, Helliwell CA (2009) Over-expression of miR172 causes loss of spikelet determinacy and floral organ abnormalities in rice (Oryza sativa). BMC Plant Biol 9: 149. 20017947 10.1186/1471-2229-9-149 PMC2803185

[38] Kagaya Y, Ohmiya K, Hattori T (1999) RAV1, a novel DNA-binding protein, binds to bipartite recognition sequence through two distinct DNA-binding domains uniquely found in higher plants. Nucleic Acids Res 27: 470–478. 9862967 10.1093/nar/27.2.470 PMC148202

[39] Chen M, Thelen JJ (2010) The plastid isoform of triose phosphate isomerase is required for the postgerminative transition from heterotrophic to autotrophic growth in Arabidopsis. Plant Cell 22: 77–90. 20097871 10.1105/tpc.109.071837 PMC2828694

[40] Raghavendra AS, Gonugunta VK, Christmann A, Grill E (2010) ABA perception and signalling. Trends Plant Sci 15: 395–401. 20493758 10.1016/j.tplants.2010.04.006

[41] Shang Y, Yan L, Liu ZQ, Cao Z, Mei C, et al (2010) The Mg-chelatase H subunit of Arabidopsis antagonizes a group of WRKY transcription repressors to relieve ABA-responsive genes of inhibition. Plant Cell 22: 1909–1935. 20543028 10.1105/tpc.110.073874 PMC2910980

[42] Finkelstein RR, Gampala SS, Rock CD (2002) Abscisic acid signaling in seeds and seedlings. Plant Cell 14 Suppl: S15–45 10.1105/tpc.010441 PMC151246 12045268

[43] Bies-Etheve N, da Silva Conceicao A, Giraudat J, Koornneef M, Leon-Kloosterziel K, et al (1999) Importance of the B2 domain of the Arabidopsis ABI3 protein for Em and 2S albumin gene regulation. Plant Mol Biol 40: 1045–1054. 10527428 10.1023/a:1006252512202

