# Supplementary figures and images for: miR172b Controls the Transition to Autotrophic Development Inhibited by ABA in Arabidopsis
Source: PLoS One. 2013 May 23;8(5):e64770. doi: 10.1371/journal.pone.0064770 (PMC3662786; doi:10.1371/journal.pone.0064770)

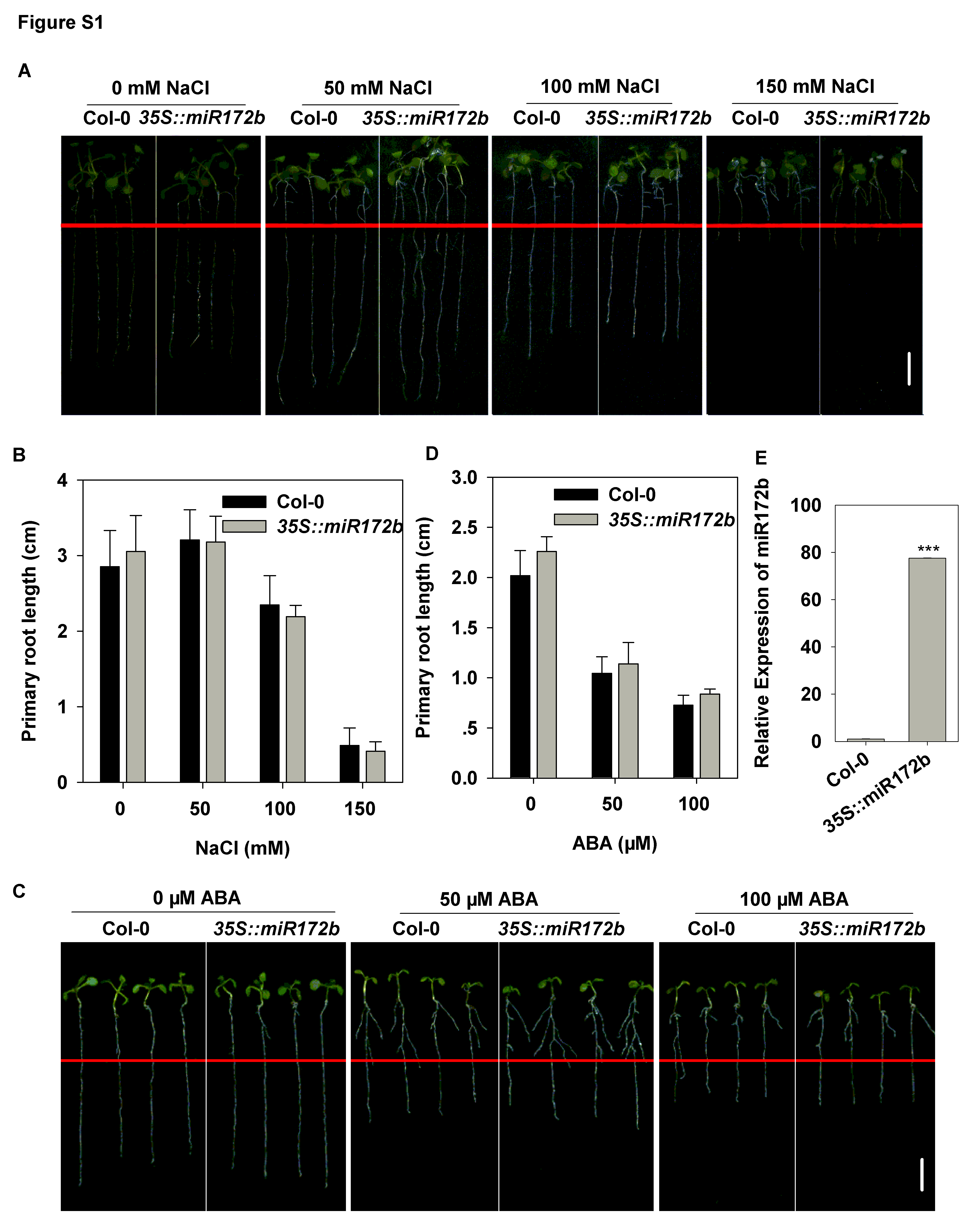

Supplement: Figure S1 — Phenotypic analysis of young seedlings of 35S::miR172b and Col-0 in response to ABA and NaCl. The seeds of 35S::miR172b and Col-0 were germinated on MS medium for 5 days and then transferred to MS medium containing NaCl (0, 50, 100 and 150 mM) or ABA (0, 50 and 100 µM). The photographs were taken 7 days after transfer to NaCl (A), and the primary root length was estimated (B). Photographs were taken 5 days after transfer to ABA (C), and the primary root length was estimated (D). (E) Identification of overexpression of miR172b in 35S:miR172b transgenic lines. Bar = 250 µm. All the experiments were performed three times with three replicates. Bars represent mean±SD. (TIF) [file pone.0064770.s001.tif]

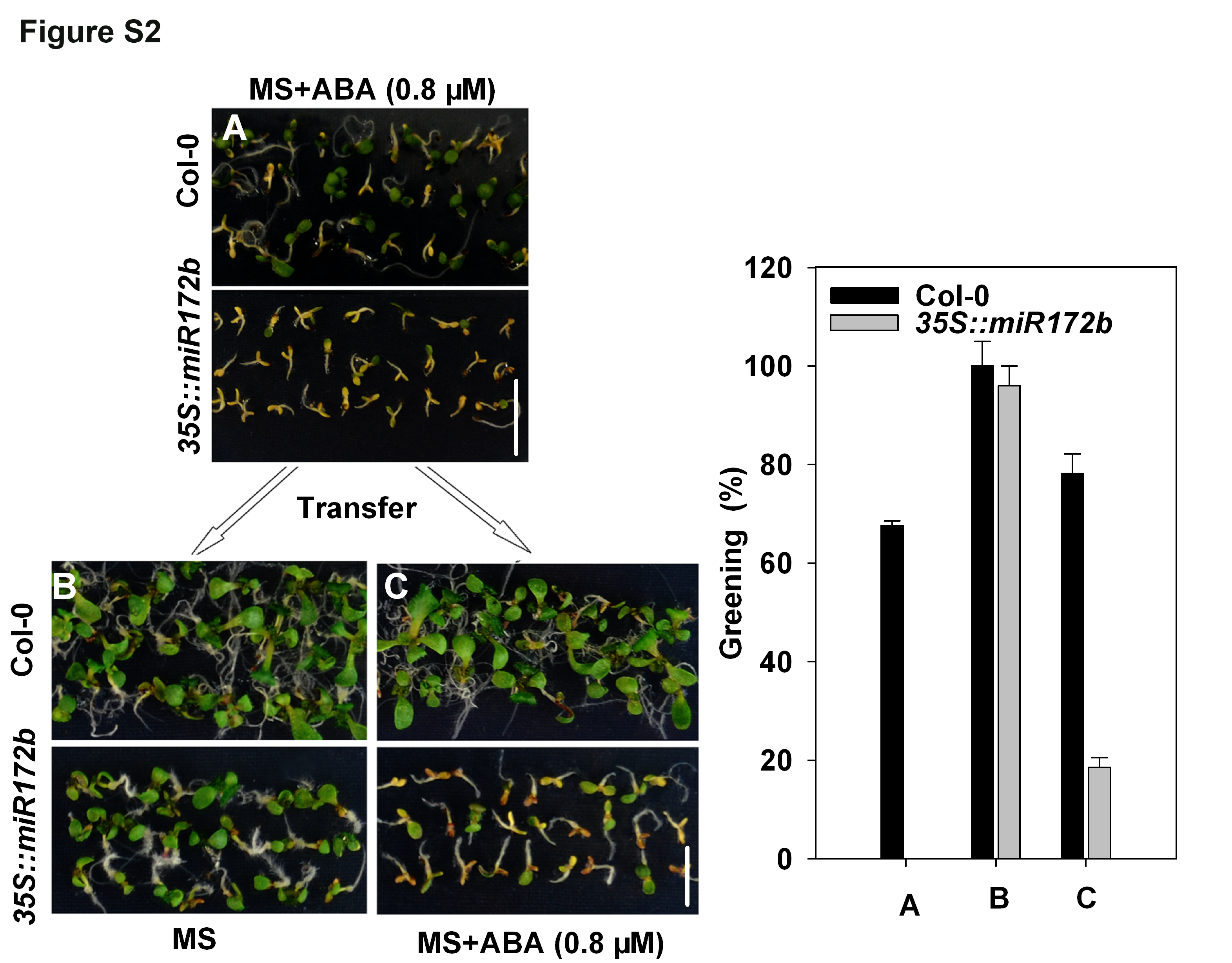

Supplement: Figure S2 — Post-geminative developmental arrest of 35S:: miR172b on ABA was restored after removing ABA. Seeds of 35::miR172b and Col-0 were germinated on MS medium containing 0.8 µM ABA, the seedlings which turned green were counted at 12 days after stratification (A); the arrested seedlings were transferred onto MS medium (B) or MS plus 0.8 µM ABA (C) for another 3 days, and the number of seedlings turning green were recorded. Bar = 5mm. All the experiments were performed three times with three replicates. Bars represent mean±SD. (TIF) [file pone.0064770.s002.tif]

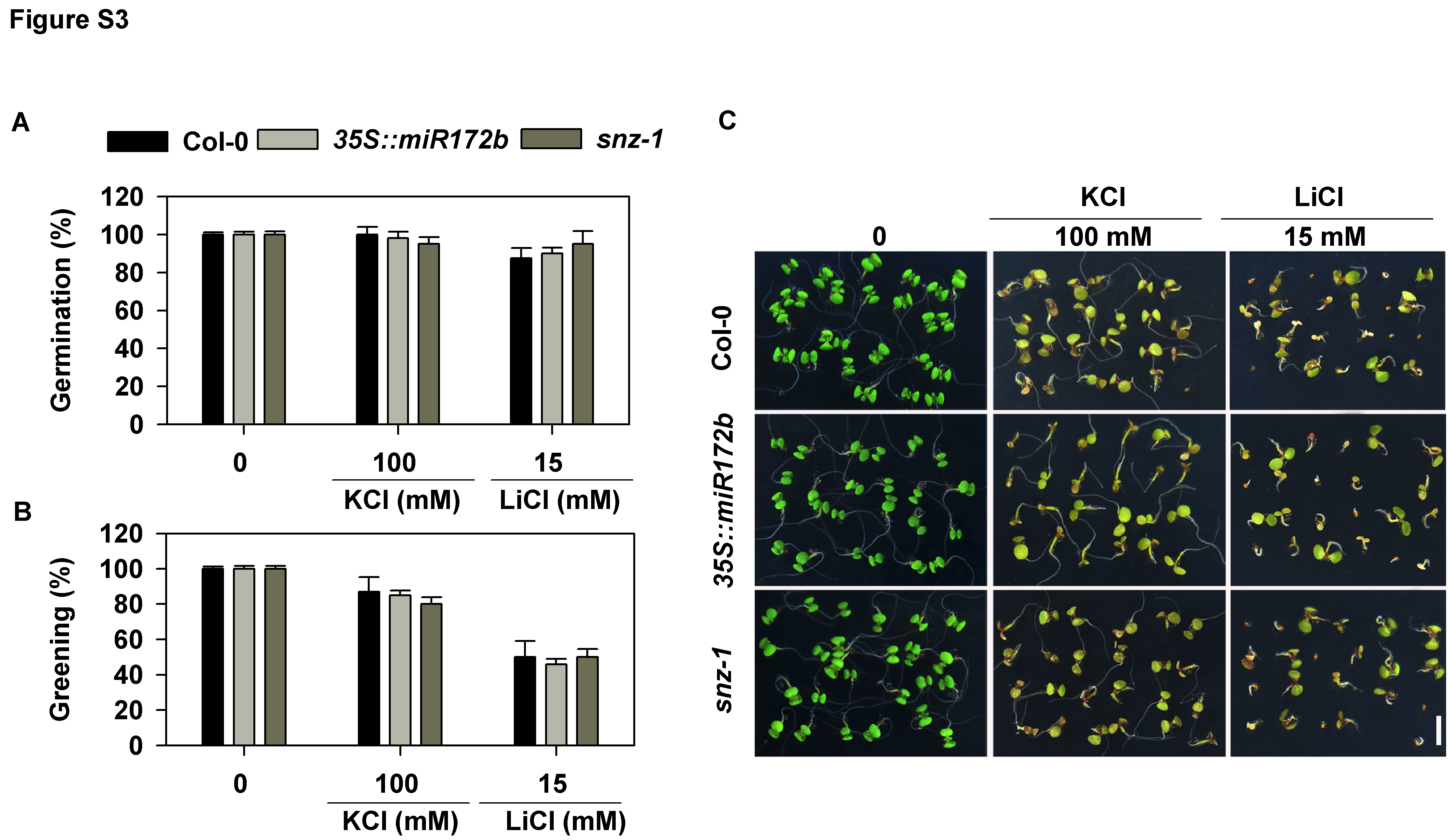

Supplement: Figure S3 — Phenotype of 35::miR172b transgenic plants and snz-1 under LiCl and KCl treatment. (A) KCl and LiCl dose–response analysis of seed germination for Col-0, 35S:: miR172b and snz-1 at 3 days after stratification. (B) and (C) Greening of 35:: miR172b transgenic plants and snz-1 was not affected under KCl and LiCl treatment. Photographs were taken 7 days after stratification. Bar = 5 mm. All the experiments were performed three times with three replicates. Error bars denote±SD. (TIF) [file pone.0064770.s003.tif]

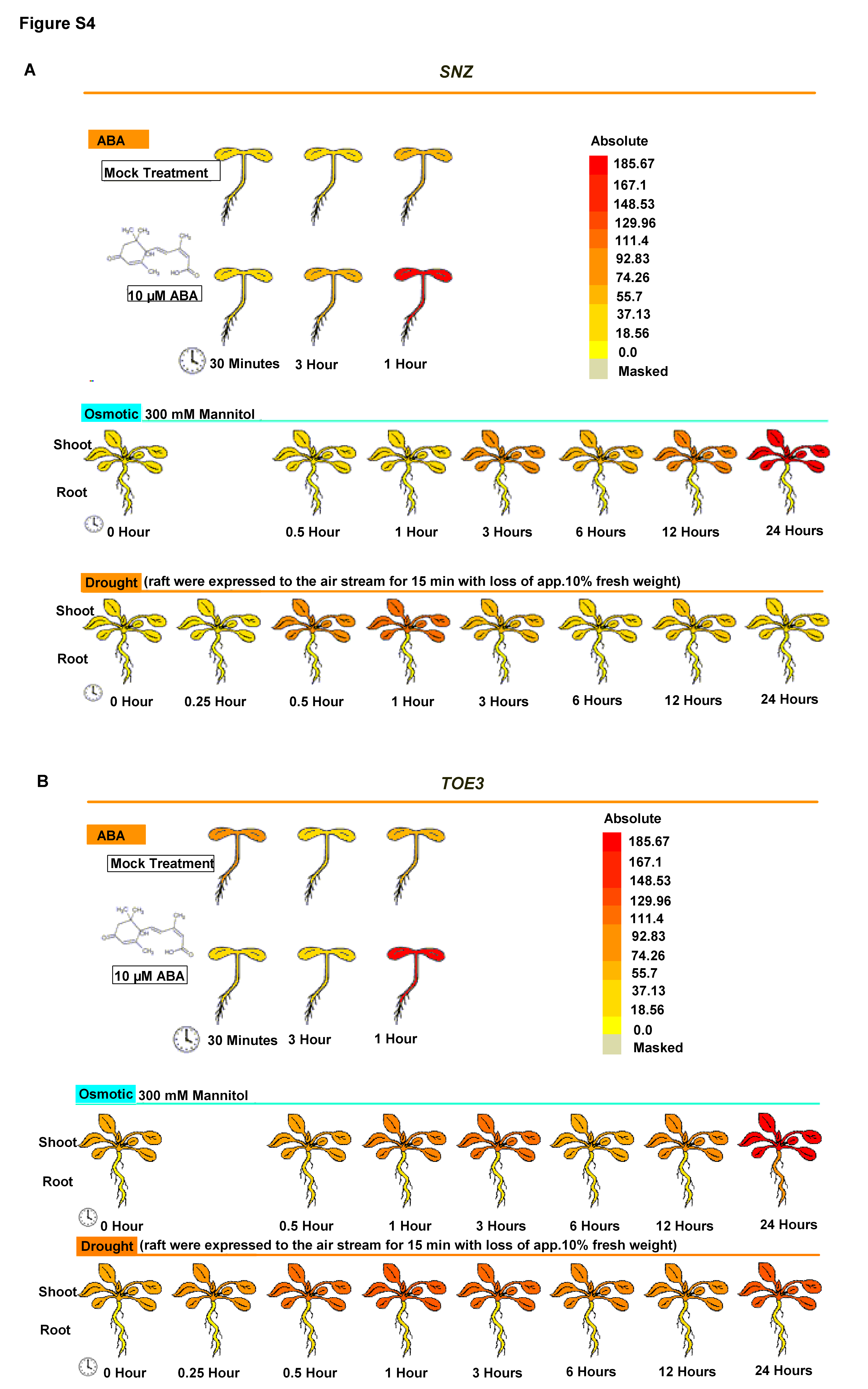

Supplement: Figure S4 — SNZ and TOE3 were up-regulated by ABA and osmotic stress/drought. Plant materials from 7 and 18 day old wild-type Col-0 was treated by ABA and osmotic stress, respectively. (A) and (B) Expression of SNZ (A) and TOE3 (B) in response to ABA and osmotic stress/drought; The colors from yellow to red indicate the increased absolute signal values of SNZ and TOE3 expression retrieved from microarray data. (http://bbc.botany.utoronto.ca/efp/cgi-bin/efpWeb.cgi). (TIF) [file pone.0064770.s004.tif]

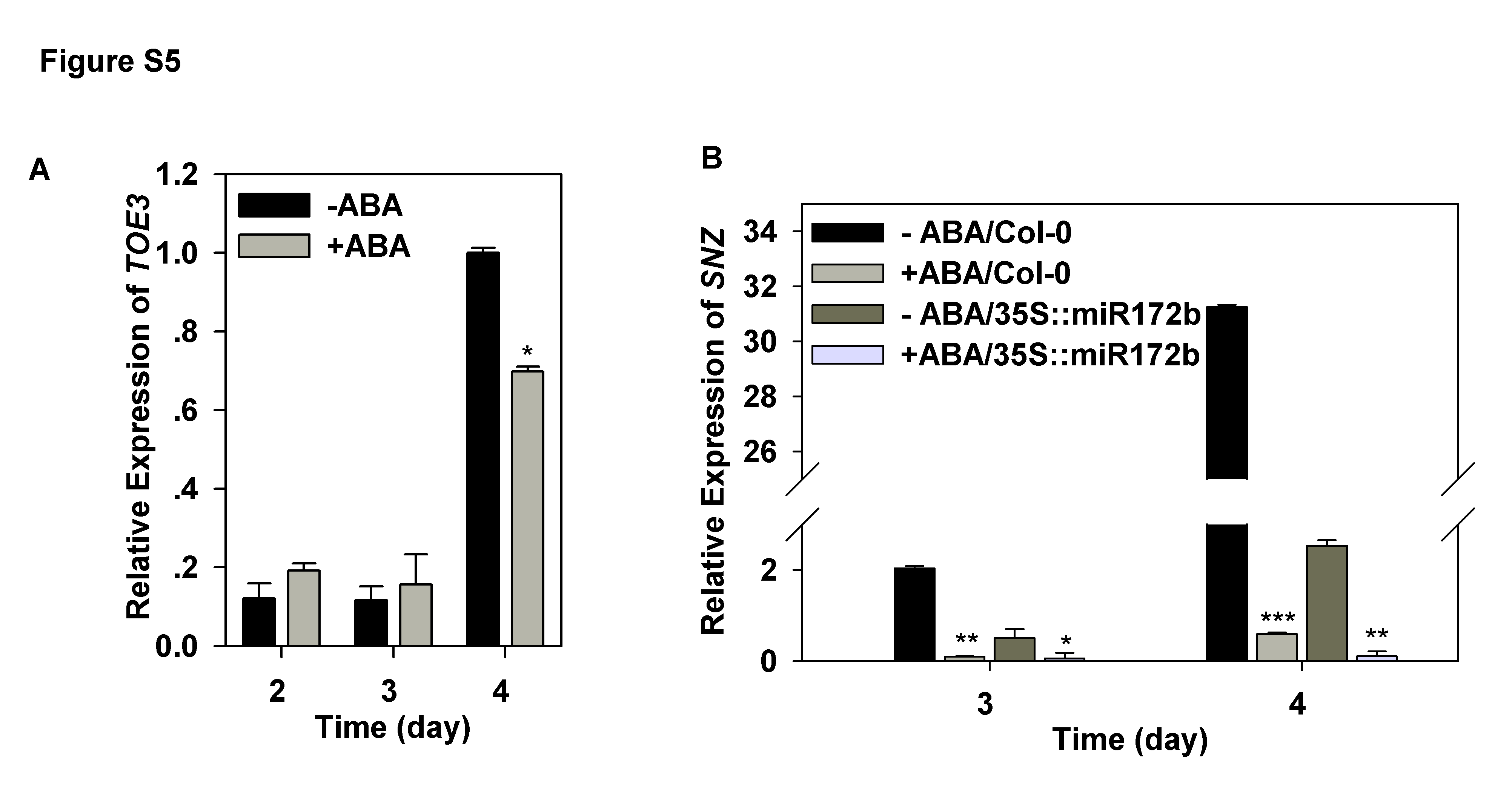

Supplement: Figure S5 — Expression of SNZ and TOE3 in response to ABA. (A) Expression analysis of TOE3 in response to ABA. Wild-type seeds were germinated on MS with or without 5 µM, and materials were collected at 2 to 4 days after stratification. (B) SNZ expression in wild-type (Col-0) and 35S:: miR172b plants on day 3 and 4 after stratification with or without 0.4 µM ABA. Student's t test was performed, and the statistically significant treatments are marked with asterisks. (*) P<0.05, (**) P<0.01 and (***) P<0.001. All the experiments were performed three times with three replicates. Error bars denote±SD. (TIF) [file pone.0064770.s005.tif]

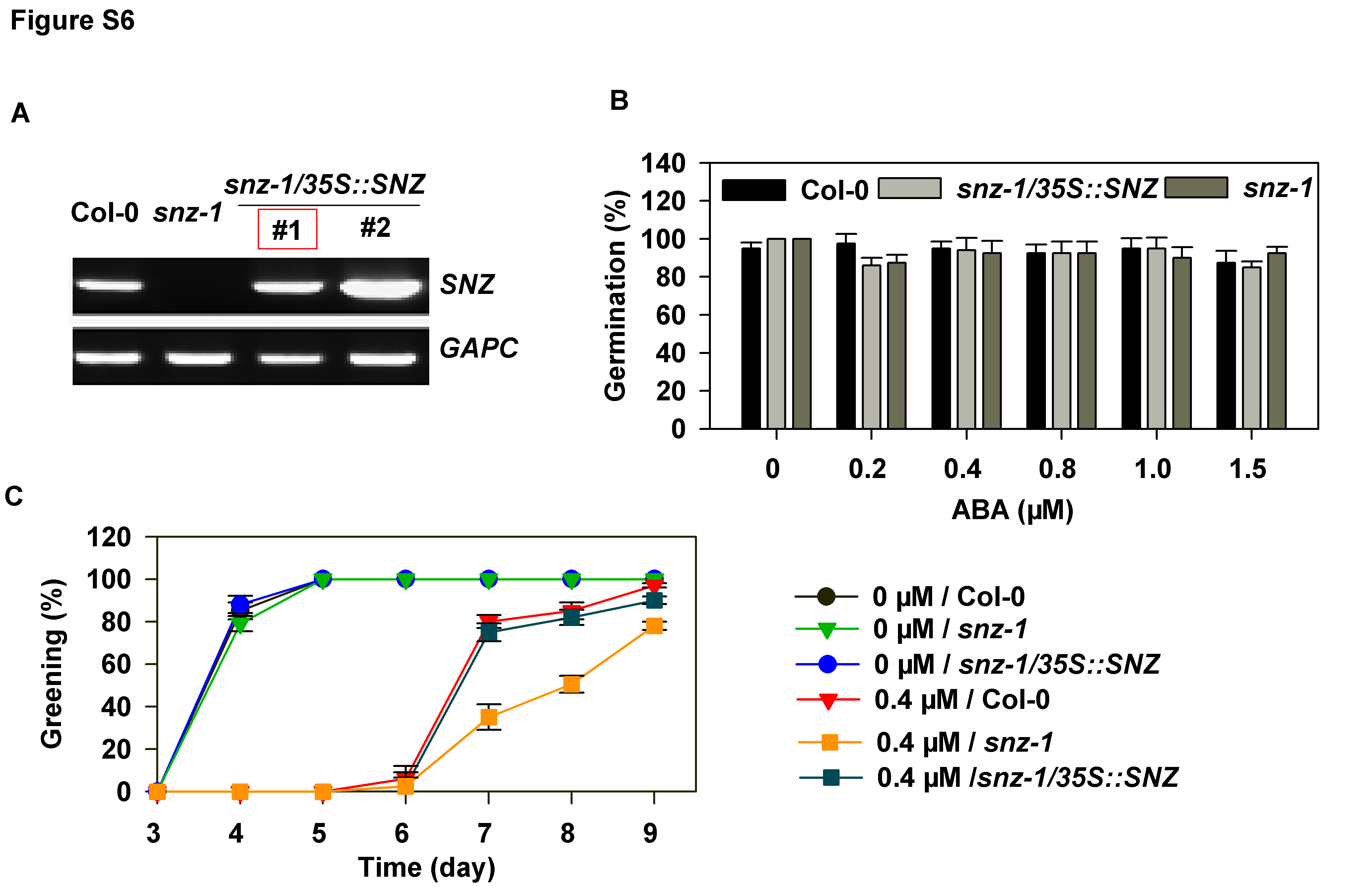

Supplement: Figure S6 — SNZ regulates post-germinative developmental arrest induced by abiotic stress through the ABA dependent pathway. SNZ cDNA under control of 35S promoter was expressed in snz-1 mutant, and the transgenic lines were characterized and used for phenotypic analysis. (A) Transcript abundance of SNZ in wild-type, snz-1 and 35S:: SNZ/snz-1 transgenic lines was monitored using RT-PCR. Shown are RT-PCR products amplified after 30 cycles, and the transcript expression levels were normalized against the reference gene GAPC. The red boxes indicate the line which was used in the phenotypic analysis. (B) ABA dose–response analysis of seed germination for Col-0 and snz-1 at 5 days after stratification. (C) A greening time course on medium containing 0.4 µM ABA. All the experiments were performed three times with three replicates. Error bars denote±SD. (TIF) [file pone.0064770.s006.tif]

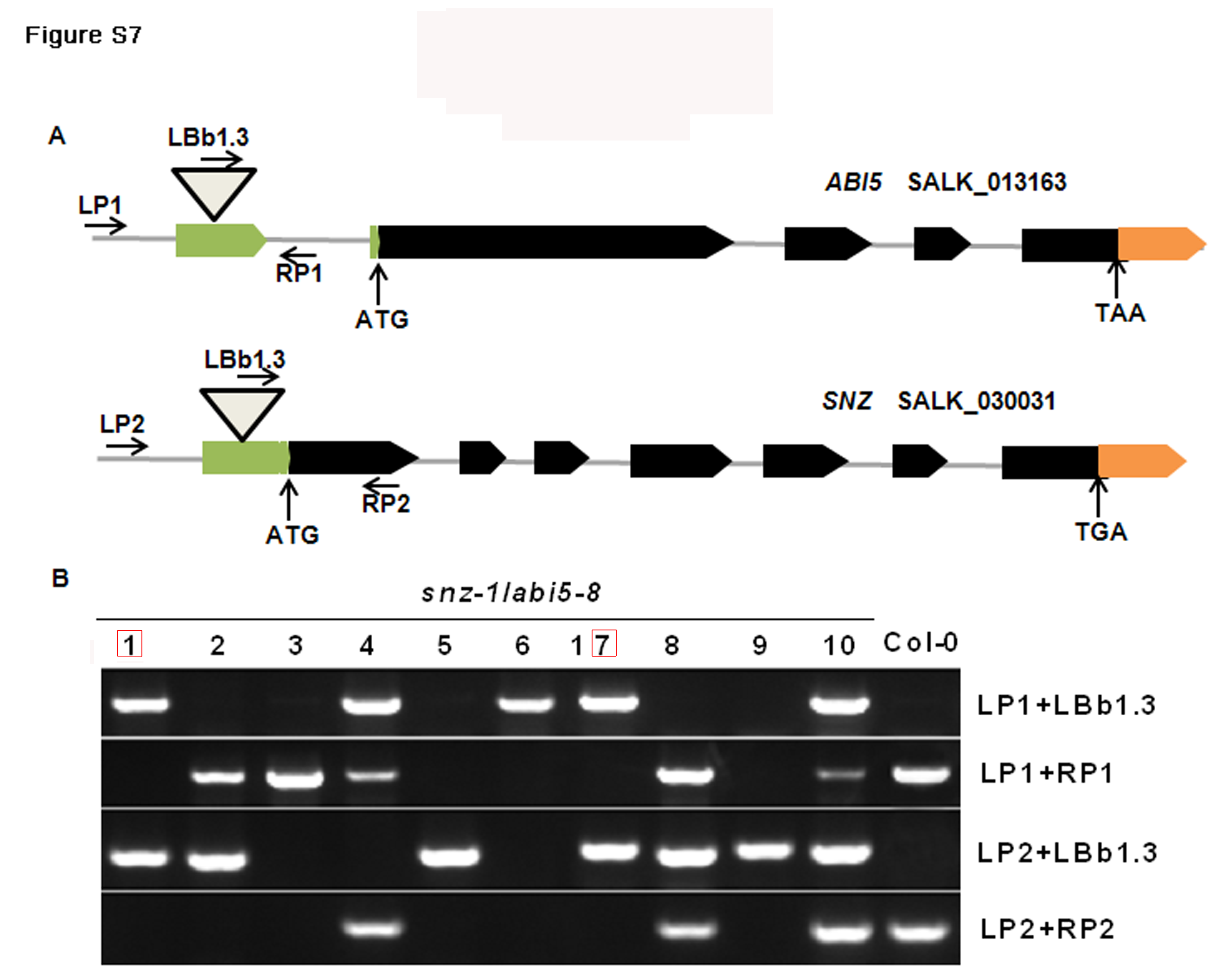

Supplement: Figure S7 — Genotyping of snz-1 / abi5-8 . (A) Schematics showing the location of the T-DNA insertion in abi5-8(SALK_013163) and snz-1(SALK_030031). Black boxes indicate exons, green box indicate 5′UTR, yellow box indicates 3′UTR, and the horizontal lines indicate introns. (B) Confirmation of the homologous T-DNA insertion in snz-1/abi5-8 by PCR. Col-0 was used as positive control. The red boxes indicate the lines with the homologous T-DNA insertions both snz-1 and abi5-8 mutations, which were used in the phenotypic analysis. (TIF) [file pone.0064770.s007.tif]
