# Supplementary material for: miR172b Controls the Transition to Autotrophic Development Inhibited by ABA in Arabidopsis
Source: PLoS One. 2013 May 23;8(5):e64770. doi: 10.1371/journal.pone.0064770 (PMC3662786; doi:10.1371/journal.pone.0064770)
Supplement: Table S1 — cis-acting regulatory elements analysis of promoter sequence of miR172b by PLACE and PROMO. (DOC) [file pone.0064770.s008.doc]

**Table S1: *cis*-acting regulatory elements analysis of promoter sequence of miR172b by PLACE and** PROMO

| **Elements** | **Sequence** | **Location** | **Reference** |
| --- | --- | --- | --- |
| **ABRE** | **ACGTG** | **400, 1600** | **Simpson et al. 2003; Nakashima et al. 2006** |
| **RY repeat** | **CATGCA** | **408** | **Ezcurra et al. 1999** |
| **Couple element 3** | **CGTGTC** | **1344, 1698** | **Hobo et al. 1999** |
| **RAV1** | **CAACA** | **993, 861** | **Kagaya et al. 1999** |
| **MYB1LEPR** | **GTTAGTT** | **1552** | **Chakravarthy et al. 2003** |
| **GAREAT** | **TAACAAR** | **1170** | **Ogawa et al. 2003** |
| **WRKY** | **TGAC** | **308, 333, 1400, 1948** | **Eulgem et al. 1999** |

**Note: The location indicate the length upstream of ‘ATG’.**

**References**

Chakravarthy S, Tuori RP, D'Ascenzo MD, Fobert PR, Despres C, et al. (2003). The tomato transcription factor Pti4 regulates defense-related gene expression via GCC box and non-GCC box cis elements. Plant Cell **15**: 3033-3050.

Eulgem T, Rushton PJ, Schmelzer E, Hahlbrock K, Somssich IE (1999). Early nuclear events in plant defence signalling: rapid gene activation by WRKY transcription factors. EMBO J **18**: 4689-4699.

Ezcurra I, Ellerstrom M, Wycliffe P, Stalberg K, Rask L (1999). Interaction between composite elements in the napA promoter: both the B-box ABA-responsive complex and the RY/G complex are necessary for seed-specific expression. Plant Mol Biol **40**: 699-709.

Nakashima K, Fujita Y, Katsura K, Maruyama K, Narusaka Y, et al. (2006). Transcriptional regulation of ABI3-and ABA-responsive genes including RD29B and RD29A in seeds, germinating embryos, and seedlings of *Arabidopsis*. Plant Mol Biol **60**: 51-68.

Hobo T, Asada M, Kowyama Y, Hattori T (1999). ACGT-containing abscisic acid response element (ABRE) and coupling element 3 (CE3) are functionally equivalent. Plant J **19**: 679-689.

Kagaya Y, Ohmiya K, Hattori T (1999). RAV1, a novel DNA-binding protein, binds to bipartite recognition sequence through two distinct DNA-binding domains uniquely found in higher plants. Nucleic Acids Res **27**: 470-478.

Ogawa M, Hanada A, Yamauchi Y, Kuwalhara A, Kamiya Y, et al. (2003). Gibberellin biosynthesis and response during *Arabidopsis* seed germination. Plant Cell **15**: 1591-1604.

Simpson SD, Nakashima K, Narusaka Y, Seki M, Shinozaki K, et al. (2003). Two different novel cis-acting elements of erd1, a clpA homologous Arabidopsis gene function in induction by dehydration stress and dark-induced senescence. Plant J **33**: 259-270.
