# Supplementary material for: miR172b Controls the Transition to Autotrophic Development Inhibited by ABA in Arabidopsis
Source: PLoS One. 2013 May 23;8(5):e64770. doi: 10.1371/journal.pone.0064770 (PMC3662786; doi:10.1371/journal.pone.0064770)
Supplement: Table S2 — cis-acting regulatory elements analysis of the SNZ promoter sequence by PLACE and AtcisDB. (DOC) [file pone.0064770.s009.doc]

**Table S2: *cis*-acting regulatory elements analysis of the *SNZ* promoter sequence by PLACE and AtcisDB**

| **Elements** | **Sequence** | **Location** | **Reference** |
| --- | --- | --- | --- |
| **ABRE** | **ACGTG** | **651** | **Simpson et al. 2003; Nakashima et al. 2006** |
| **ATHB2** | **TAATCATTA** | **211** | **Steindler et al. 1999** |
| **DPBF** | **ACACATG** | **35, 590, 925** | **Kim et al. 1997; Finkelstein and Lynch 2000; Lopez-Molina and Chua 2000; Nakashima et al. 2006** |
| **RAV1** | **CAACA** | **646, 150** | **Kagaya et al. 1999** |

**Note: The location indicates the length upstream of ‘ATG’.**

**References**

Finkelstein RR, Lynch TJ (2000). The Arabidopsis abscisic acid response gene ABI5 encodes a basic leucine zipper transcription factor. Plant Cell **12**: 599-609.

Kagaya Y, Ohmiya K, Hattori T (1999). RAV1, a novel DNA-binding protein, binds to bipartite recognition sequence through two distinct DNA-binding domains uniquely found in higher plants. Nucleic Acids Res **27**: 470-478.

Kim SY, Chung HJ, Thomas TL (1997). Isolation of a novel class of bZIP transcription factors that interact with ABA-responsive and embryo-specification elements in the Dc3 promoter using a modified yeast one-hybrid system. Plant J **11**: 1237-1251.

Lopez-Molina L, Chua NH (2000). A null mutation in a bZIP factor confers ABA-insensitivity in Arabidopsis thaliana. Plant Cell Physiol **41**: 541-547.

Nakashima K, Fujita Y, Katsura K, Maruyama K, Narusaka Y, et al. (2006). Transcriptional regulation of ABI3-and ABA-responsive genes including RD29B and RD29A in seeds, germinating embryos, and seedlings of *Arabidopsis*. Plant Mol Biol **60**: 51-68.

Simpson SD, Nakashima K, Narusaka Y, Seki M, Shinozaki K, et al. (2003). Two different novel cis-acting elements of erd1, a clpA homologous Arabidopsis gene function in induction by dehydration stress and dark-induced senescence. Plant J **33**: 259-270.

Steindler C, Matteucci A, Sessa G, Weimar T, Ohgishi M, et al. (1999). Shade avoidance responses are mediated by the ATHB-2 HD-zip protein, a negative regulator of gene expression. Development **126**: 4235-4245.
