# Supplementary material for: miR172b Controls the Transition to Autotrophic Development Inhibited by ABA in Arabidopsis
Source: PLoS One. 2013 May 23;8(5):e64770. doi: 10.1371/journal.pone.0064770 (PMC3662786; doi:10.1371/journal.pone.0064770)
Supplement: Table S3 — Primers pairs used for real-time RT-PCR (Sequence 5′→3′). (DOC) [file pone.0064770.s010.doc]

**Table S3: Primers pairs used for real-time RT-PCR (Sequence 5’→3’)**

| **Primer** | **Sequence（F）( 5’→3’)** | **Sequence（R）( 5’→3’)** | **Reference** |
| --- | --- | --- | --- |
| **qmiR172b** | **TTTCTCAAGCTTTAGGTATTTGTAG** | **TCGGCGGATCCATGGAAGAAAGCTC** | **Wu et al. 2009** |
| ***qABI3*** | **CGGGAGGGACCTGGATGTATT** | **CCATCACTGGCGGTAATTGAG** | **Xi et al. 2010** |
| ***qABI5*** | **CAGCTGCAGGTTCACATTCTG** | **CACCCTCGCCTCCATTGTTAT** | **Xi et al. 2010** |
| ***qEm1*** | **CGGAGGAAGAAGGGATTGAGA** | **TGCCAAACACGGAACCTACA** | **Jiang et al. 2009** |
| ***qEm6*** | **AGGATATCAGCAGATGGGACGC** | **CGTCTATCTCGACTCCTTCCTC** | **Xi et al. 2010** |
| ***qRAB18*** | **TGGCTTGGGAGGAATGCTTCA** | **CCATCGCTTGAGCTTGACCAGA** | **Saez et al. 2008** |
| ***qMFT*** | **CGAGCCGAACATGAGAGAAT** | **AAGTATCTCTTTTCCTCTTGAGGG** | **Xi et al. 2010** |
| ***qSNZ*** | **CGACACACAACGAAGATGA** | **GGGAGGAGGAAGGATTTC** | **This work** |
| ***qTOE3*** | **AAGAATCCGACAGTAGAGGG** | **CTTACCGACACTATTGAAACCG** | **This work** |
| ***qGAPC*** | **TTGGTGACAACAGGTCAAGCA** | **AAACTTGTCGCTCAATGCAAT** | **Czechowski et al. 2005** |

**References**

Czechowski T, Stitt M, Altmann T, Udvardi MK, Scheible WR (2005). Genome-wide identification and testing of superior reference genes for transcript normalization in *Arabidopsis*. Plant Physiol **139**: 5-17.

Jiang W, Yu D (2009) Arabidopsis WRKY2 transcription factor mediates seed germination and postgermination arrest of development by abscisic acid. BMC Plant Biol 9: 96.

Saez A, Rodrigues A, Santiago J, Rubio S, Rodriguez PL (2008) HAB1-SWI3B Interaction Reveals a Link between Abscisic Acid Signaling and Putative SWI/SNF Chromatin-Remodeling Complexes in Arabidopsis. Plant Cell 20: 2972-2988.

Wu G, Park MY, Conway SR, Wang JW, Weigel D, et al. (2009). The sequential action of miR156 and miR172 regulates developmental timing in *Arabidopsis*. Cell **138**: 750-759.

Xi W, Liu C, Hou X, Yu H (2010). MOTHER OF FT AND TFL1 regulates seed germination through a negative feedback loop modulating ABA signaling in Arabidopsis. Plant Cell **22**: 1733-1748.
