# Supplementary material for: miR172b Controls the Transition to Autotrophic Development Inhibited by ABA in Arabidopsis
Source: PLoS One. 2013 May 23;8(5):e64770. doi: 10.1371/journal.pone.0064770 (PMC3662786; doi:10.1371/journal.pone.0064770)
Supplement: Table S4 — Primers pairs used for construction and genotyping, probes used for EMSA (Sequence 5′→3′). (DOC) [file pone.0064770.s011.doc]

**Table S4: Primers pairs used for construction and genotyping, probes used for EMSA (Sequence 5’→3’)**

| **Primer** | **Sequence ( 5’→3’)** |
| --- | --- |
| **LP1** | **CAATGGAAGTTCGGAATCATG** |
| **RP1** | **CACTCGTTTTCTTCTTAAAGCG** |
| **LP2** | **TCCCCTCCACATACAAAAGTG** |
| **RP2** | **AAATATGAGATTCCCAACGGC** |
| **LBb1.3** | **ATTTTGCCGATTTCGGAAC** |
| **35S::SNZ-XbaⅠ-F** | **GCTCTAGA ATGTTGGACCTCAACCTCGG** |
| **35S::SNZ-SacⅠ-R** | **CGAGCTC TCATGGATCAAAACAAGATAAG** |
| **wRAV1-A probe** | **cacctg(CAACA)_6_cacctg** |
| **mRAV1-A probe** | **cacctg(CGGTA)_6_cacctg** |
| **ABI5 probe** | **ttaacaactgcatcatatacaCAACAataac** |
