# Supplementary material for: miR172b Controls the Transition to Autotrophic Development Inhibited by ABA in Arabidopsis
Source: PLoS One. 2013 May 23;8(5):e64770. doi: 10.1371/journal.pone.0064770 (PMC3662786; doi:10.1371/journal.pone.0064770)
Supplement: Table S5 — Information of the plant materials used in this work. (DOC) [file pone.0064770.s012.doc]

**Table S5: Information of the plant materials used in this work**

| **Gene** | **Materials** | **Gene ID** | **SALK line** | **Providers** |
| --- | --- | --- | --- | --- |
| **miR172b** | ***35S::miR172b*** | **AT5G04275** |  | **R. Scott Poethig** |
| ***SNZ*** | ***snz-1*** | **AT2G39250** | **SALK_030031** | **Markus Schmid** |
| ***ABI5*** | ***abi5-8*** | **AT2G36270** | **SALK_013163** | **This work** |
