# Supplementary material for: miR172b Controls the Transition to Autotrophic Development Inhibited by ABA in Arabidopsis
Source: PLoS One. 2013 May 23;8(5):e64770. doi: 10.1371/journal.pone.0064770 (PMC3662786; doi:10.1371/journal.pone.0064770)
Supplement: Table S6 — cis-acting regulatory elements RAV1 analysis of promoter sequence of ABA response genes by PLACE and AtcisDB. (DOC) [file pone.0064770.s013.doc]

**Table S6: cis-acting regulatory elements RAV1 analysis of promoter sequence of ABA response genes by PLACE and AtcisDB**

| **Gene** |  | **Element** | **Sequence** | **Number of the element** |
| --- | --- | --- | --- | --- |
| ***ABI3*** | ***AT3G24650*** | **RAV1-A** | **CAACA** | **8** |
| ***ABI5*** | ***AT2G36270*** | **RAV1-A** | **CAACA** | **13** |
| ***RAB18*** | ***AT1G43890*** | **RAV1-A** | **CAACA** | **11** |
| ***MFT*** | ***AT1G18100*** | **RAV1-A** | **CAACA** | **13** |
| ***Em1*** | ***AT3G51810*** | **RAV1-A** | **CAACA** | **9** |
| ***Em6*** | ***AT2G40170*** | **RAV1-A** | **CAACA** | **7** |

**Note: A 2 kb sequence upstream of ‘ATG’ for each gene was analyzed.**
